# Citizen science-based biodiversity monitoring in oil palm plantations

**DOI:** 10.1371/journal.pone.0318046

**Published:** 2026-08-25

**Authors:** Nunik Maharani, N Nardiyono, Claudia Retina Munthe, Priya Swayanuar, Safwanah Ni’Matullah, Syafiie Sueif, Syahmi Zaini, Jatna Supriatna, Mirza Kusrini, Rona Dennis, Bas van Balen, Arco van Strien, Erik Meijaard

**Affiliations:** 1 Austindo Nusantara Jaya, Jakarta, Indonesia; 2 Borneo Futures, Bandar Seri Begawan, Brunei Darussalam; 3 Department of Biology, Faculty of Mathematics and Natural Sciences, Universitas Indonesia, Depok, Indonesia; 4 Institute for Sustainable Earth and Resources (I-SER), Depok, Indonesia; 5 Dept. of Forest Resources Conservation & Ecotourism, IPB University, Darmaga, Indonesia; 6 Basilornis, Arnhem, the Netherlands; 7 Arco van Strien Data Analysis, Leiden, the Netherlands; 8 Institute for Advanced Studies, University of Amsterdam, Amsterdam, The Netherlands; University of Queensland, AUSTRALIA

## Abstract

Agricultural expansion is one of the main threats to global biodiversity. Yet, many wildlife species survive, and sometimes even thrive, in agricultural landscapes that retain patches of natural ecosystems. This paradox is evident in tropical oil palm (*Elaeis guineensis*, Jacq.) plantations that have both replaced tropical forest and other species-rich ecosystems, but can also function as wildlife habitat, especially if fragments of natural ecosystems are retained. There is an urgent need to understand how to manage and monitor wildlife in these landscapes. The lack of large, quantitative datasets on species occurrence, however, impedes learning and adaptive management. To address this gap, we piloted a citizen science-based biodiversity monitoring system in seven Indonesian oil palm estates, across different biogeographical regions, over a 5-year period. Between September 2019 and June 2024, contributions from 3,950 company employees resulted in 148,286 wildlife observations of 699 reliably identified faunal and 186 floral species. This is, to our knowledge, the first biodiversity monitoring system of its kind in the oil palm sector. Management support at all levels of the company, together with an employee reward system, proved vital for its implementation. We demonstrate how these observations can be used to estimate species occupancy and quantify wildlife use across heterogeneous plantation landscapes. Monitoring costs ranged from USD 0.14/ha/y to USD 0.32/ha/y, substantially lower than those of many conventional wildlife survey approaches. We conclude that citizen science can provide a robust, scalable and cost-effective approach in oil palm plantations, generating the quantitative data needed to support adaptive management.

## Introduction

Oil palm is one of the world’s most important vegetable oil crops, covering 24 million hectares in the species-rich tropics, but producing more oil than any other crop [1]. At the same time, oil palm cultivation has become central to global debates on biodiversity decline because of its role in replacing tropical forests and other natural ecosystems [2,3]. Under pressure, most notably from non-government organizations and consumers, the palm oil industry has faced increasing pressure to improve its environmental performance. With the establishment of the Roundtable on Sustainable Palm Oil (RSPO) in 2004, there has been some progress in reducing negative impacts on biodiversity in plantation landscapes [4–6], through biodiversity management requirements such as setting aside areas with High Conservation Values and by avoiding deforestation in plantation development [7]. Determining whether such measures actually benefit the diversity and abundance of wildlife in certified plantations requires measurable indicators and their monitoring [8].

The detailed level of information necessary for effectively monitoring the impacts of plantation management practices on biodiversity necessitates expansive and frequent data collection [9]. This is, however, costly and requires expertise that many plantation companies lack. The uptake of quantitative monitoring of the diversity and abundance of wildlife in and around oil palm plantations has therefore been slow [10]. Consequently, most companies rely on annual species lists for monitoring purposes, primarily focusing monitoring efforts on forest areas, not across planted oil palm [6]. Adaptive management at the level of the entire estate based only on species presence information is, however, difficult. Instead, quantitative information at the population level and across the entire landscape is needed to inform management interventions [11]. In the complex landscapes of oil palm plantations, the need for innovative approaches to monitor and manage biodiversity is urgent [9].

Conventional biodiversity monitoring methods are often constrained by logistical, financial and technical challenges, especially in large agricultural landscapes, necessitating innovative solutions to enhance data acquisition and analysis [10]. Citizen science, defined as the participation of non-professional volunteers in scientific research [12], has emerged as an approach for expanding the scope and scale of ecological data collection [13]. By engaging large numbers of observers, citizen science can generate datasets that would be prohibitively expensive or impractical to obtain using conventional survey methods alone. In biodiversity conservation, it has proven valuable for documenting species distributions, monitoring population trends, detecting rare species, and supporting species distribution and occupancy modelling [14].

Studies have shown that, with appropriate training and quality assurance procedures, citizen science can yield data of comparable quality to those collected with traditional scientific methods [15]. Nevertheless, the integration of citizen science data with quantitative ecological analyses presents important challenges, including ensuring data reliability, accounting for variation in observer skill, and addressing spatial and temporal sampling biases [16]. These challenges are particularly relevant where monitoring data are intended to inform adaptive management and decision-making in production landscapes. Unlike many citizen science initiatives that rely on members of the public, our approach leverages plantation employees who are already distributed throughout the landscape as part of their routine work, creating opportunities for continuous, landscape-scale biodiversity monitoring.

Starting in 2019, our pilot project in the Indonesian palm oil company Austindo Nusantara Jaya (ANJ) tested whether citizen science-based biodiversity monitoring could be effectively implemented in oil palm estates. Specifically, we assessed whether the approach could generate statistically robust wildlife occurrence data, and whether these data could support an adaptive wildlife management cycle. We also evaluated the organisational and social aspects of implementing a citizen science approach. Details of the statistical methods are presented in a companion paper in this journal [16], and the social dimensions of the program elsewhere [17].

This paper examines the role of citizen science in addressing the biodiversity monitoring and management challenges specific to oil palm plantations, with a focus on practitioners who require practical guidance for meeting regulatory wildlife monitoring requirements. Specifically, we address four questions: 1) What organizational factors enabled successful implementation of the program? 2) What was the uptake of the program by palm oil workers as expressed as percentage of total work force? 3) Can a citizen science approach generate estate-wide data collection from a wide range of reliably identified species, and do the data include species of conservation concern? and 4) How do the costs of citizen science-based monitoring compare with more conventional, technology-intensive approaches?

## Methods

### Securing company commitment

#### Project design.

Leading up to project initiation, there were extensive discussions with company senior management. A pilot implementation in 2017 did not result in sustained participation. A review conducted before the second implementation suggested that limited engagement by estate management, insufficient communication of the project’s objectives, and inadequate training contributed to the lack of uptake. This highlighted the importance of securing support from senior company management before implementation. Prior to commencing the program in 2019, a series of meetings was held with senior management to explain the objectives of the citizen science approach, expected staff commitments, anticipated conservation benefits, and management responsibilities.

Although participation by individual employees in recording wildlife observations was voluntary, senior ANJ management required estate managers to facilitate implementation of the PENDAKI program within their estates, with responsibility for implementation incorporated into Key Performance Indicators for specific staff. Furthermore, the company implemented a non-monetary rewards program that gave small tokens of appreciation (e.g., T-shirts, caps, and raincoats) to observers for the most interesting wildlife observations, or recognised them in sustainability reports, the company website and internal newsletters.

### Project initiation and location

The program was initiated through a one-week in-person training in 2019 by RD for 2 conservation managers and 12 conservation and sustainability compliance staff. In the same week, BvB gave a parallel training on bird identification. The citizen science training introduced the principles of citizen science and encouraged participants to develop implementation strategies tailored to the operational context of their respective estates and staff capacity. Participants also selected the project name, resulting in the name “PENDAKI”, which is abbreviated Indonesian for Caring for Biodiversity (*Peduli Keanekaragaman Hayati*). Through this approach, conservation staff were positioned as the primary drivers of project conceptualization and implementation the project, with external advisors serving a facilitative rather than a leading role. In 2023, RD conducted a second training session to standardize data collection methods. AvS subsequently provided additional training in data management, occupancy analysis, and statistical analysis of wildlife observations in the R software environment [18].

**Fig 1:**
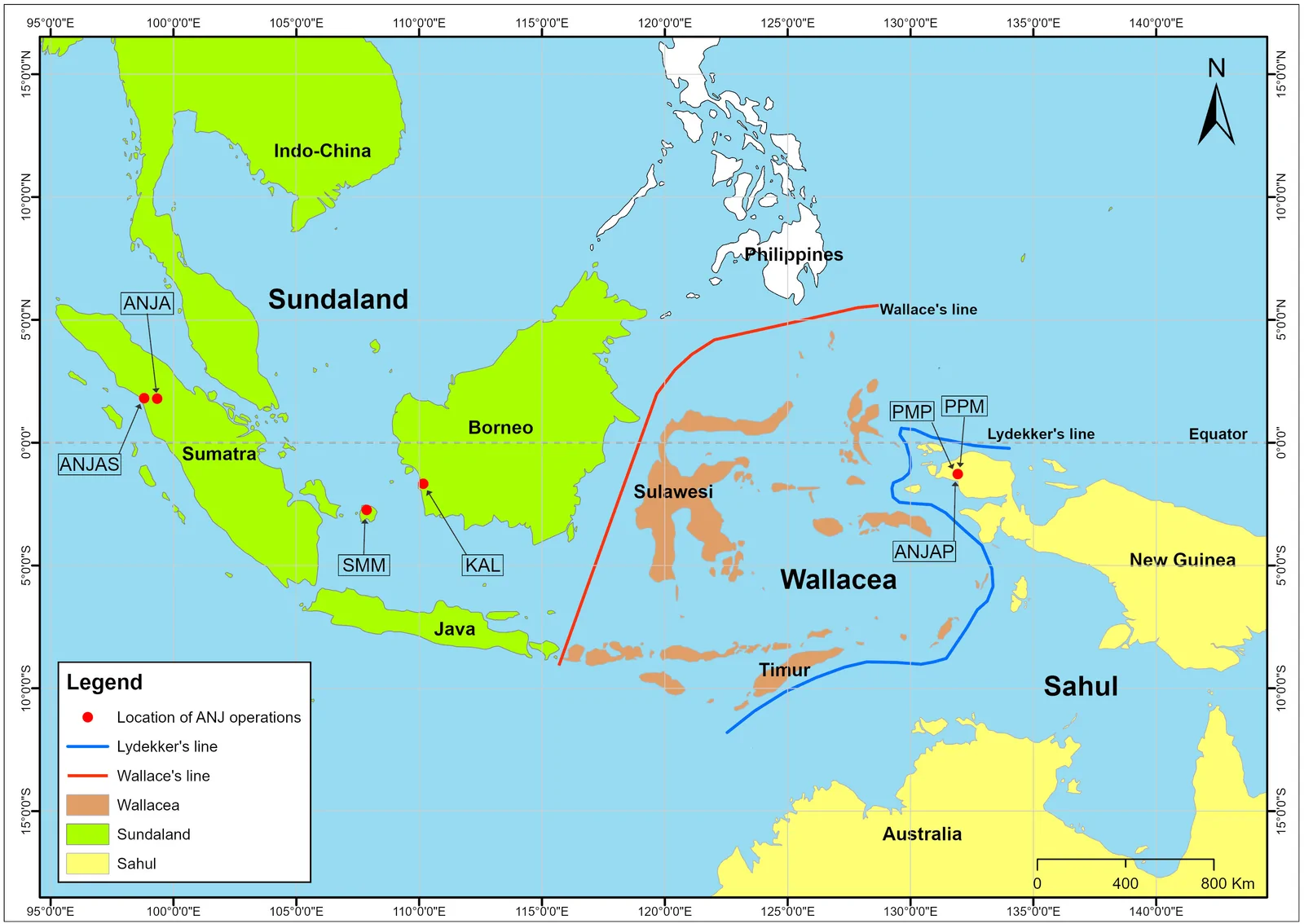
Location map of the 7 Indonesian estates. Abbreviated estate names show where the PENDAKI program was implemented. Also shown are the two biogeographic regions and the Wallace and Lydekker’s lines separating them. Basemap is from.

The citizen science data collection started in September 2019 in six oil palm estates in Sumatra (ANJA and ANJAS), Belitung (SMM), Kalimantan (KAL) and Southwest Papua (PMP, PPM) (Fig 1), and one sago (*Metroxylon sagu*, Rottb.) concession (ANJAP). The combined total area of these estates is 144,611 ha, of which 59,867 ha (41%) had been set aside by the company for biodiversity conservation purposes and maintenance of High Conservation Value (HCV) areas [19]. The estates spanned two distinct biogeographic regions, Sundaland and Sahul, encompassing very different floras and faunas (Fig 1).

### Participation by estate workers and data collection

Participation in PENDAKI was open to any company employee, contractor, or external visitor who volunteered to report wildlife observations. Participants included core conservation staff, employees whose work was unrelated to biodiversity monitoring and management (e.g., harvesters, mill workers, drivers, security guards, cooks, cleaners, estate managers), as well as external visitors such as journalists, biodiversity consultants, and government officials. Wildlife observations could be reported by recording them on a paper form, or directly reported to the conservation units in each estate (either through in-person meetings or mobile phone communication). Each conservation unit at each estate developed local WhatsApp groups, which provided support with species identification and helped sustain participation.

In 2023, a custom-built smartphone application, PENDAKI Champion, was rolled out across six oil palm estates to increase data collection and to link each wildlife observation to a precise geographic coordinate. The application included a list of 25 target species for each estate, and PENDAKI Champion users were encouraged to record observations whenever these target species were encountered.

We estimated the total number of PENDAKI participants by identifying unique names associated with submitted wildlife observations. Minor manual reconciliation was involved due to inconsistent spelling of observer names, which occasionally made it difficult to reliably link records to a single individual.

### Interview surveys on program uptake and perceptions

To better understand the uptake of PENDAKI and how this was perceived by its users and company management, RD conducted semi-structured interviews with employees and management. In June 2022, 39 staff members were interviewed across the six ANJ oil palm subsidiaries and the head office (Supplementary Materials), with five or six participants interviewed from each location. Interviewees were selected by ANJ staff to provide representation across subsidiaries and staff roles. A semi-structured interview comprising 13 questions was used (S1 Table), and each interview lasted about 20 minutes, with the interview being recorded (S2 File Supporting Methods).

RD and EM conducted a follow-up evaluation in June 2024 to assess the functioning of the PENDAKI Champion smartphone app as well as the original PENDAKI system. Focus group discussions were held in four estates (ANJA, ANJAS, PPM, and PMP) with the conservation managers, 30 PENDAKI Champion app users (all male) and 12 original PENDAKI users (four female). Each focus group lasted approximately 3 hours and explored participants’ experiences of using the system, perceived strengths and weaknesses, technical and implementation challenges, and opportunities for improvement.

### Data management, validation, and cleaning

The PENDAKI program was initially paper based with each individual wildlife observation being recorded on a form, with all the relevant details of the observation (date and time, location, species name, and behaviour of species) and a photo, if available. This information was then entered monthly into an Excel spreadsheet, and each estate shared these sheets with the head office in Jakarta, where the data was compiled into the master database. This approach deliberately placed responsibility for data collection and entry with the estate staff, with the aim of fostering a sense of ownership over the program and data. In 2021, ANJ developed an internet-based dashboard, linked to the master database. The dashboard allowed database managers at the estate-level to consult the status of submitted observations (verified and approved or rejected) in near real-time.

In 2023, the PENDAKI smartphone application was introduced through a phased roll-out beginning with the 10 most active data collectors per estate (‘PENDAKI Champions’). The paper-based and application-based system ran in parallel with all non-app data being compiled and uploaded to the PENDAKI database.

Wildlife observation data were stored in the PENDAKI database set up by ANJ in their Jakarta head-office. ANJ data managers (CRM and PS) did the initial cleaning of records, standardizing species names, and deleting records that were obviously erroneous. The resulting clean database was regularly downloaded by the data reviewers. The PENDAKI database could be accessed by all estates through a dashboard that listed the individual species observations and provided constantly updated summary information (e.g., number of monthly observations per estate or per observer, records requiring verification, and running totals for different species groups).

On an annual basis, independent species experts (authors EM, RD, BvB and SS) validated the PENDAKI records and determined the likelihood of the reported wildlife sightings being correct using the following categories: Likely Correct; Unlikely but Possibly Correct; Probably Incorrect; Correct ID but Wrong Species Name; Very Difficult to Identify; and Not Identified at Species Level (for definitions see S3 Table). The reviews used species photographs, and knowledge of species distribution and most recent taxonomy to determine the accuracy of records. Following each data review, a report was provided to the company with key species identification issues, for example, species that were consistently misidentified (e.g., Chinese Egret *Egretta eulophotes*, instead of Little Egret *Egretta garzetta*).

We designed the data collection methods, and especially the PENDAKI Champion application, to facilitate analysis using occupancy models, which are ideally suited for the opportunistic, non-structured wildlife observations to determine spatial and temporal changes for species [20,21]. Occupancy models estimate the proportion of sites occupied by a species while accounting for imperfect detection [16]. Occupancy modelling separates two questions that are easy to confuse in unstructured wildlife data: “was the species actually present at a site?” and “did an observer happen to notice it?” A species can go unrecorded at a site simply because no one looked hard enough, not because it was truly absent. Our models addressed this by using each site’s repeated visits over a year to estimate, for every species, both (i) the probability that the site was occupied, and (ii) the probability that an observer would detect the species given that it was present. This detection probability was allowed to vary with the number of other species reported on the same visit (a proxy for how much effort or attention an observer put into that visit), with whether the sighting was made in forest or non-forest habitat, and with the identity of the individual observer, since some people are more likely to notice and report wildlife than others. By modelling detection separately from true occupancy, we could estimate how much of the estate each species used without that estimate being distorted by uneven effort, observer skill, or habitat-related differences in how easy a species is to spot. For technical details and model assumptions refer to the companion paper [16].

As an illustration, we provide spatial and temporal occupancy (or distribution) trends of orangutan (*Pongo pygmaeus*), a species normally only associated with forest habitats, but increasingly understood to also survive in heterogenous landscape of forest and agriculture, such as oil palm [22–24]. We also present an occupancy analysis for the White-breasted Waterhen (*Amaurornis phoenicurus*), a common bird species in planted oil palm, illustrating the applicability of the approach to both forest-dependent and plantation-associated wildlife.

### Cost analysis and comparison

We compared the costs of the PENDAKI monitoring system with those of other wildlife monitoring approaches to assess the relative costs for large-scale biodiversity monitoring in oil palm plantations. Implementation costs were estimated by collecting information on staffing costs, costs of any materials used in project implementation (e.g., rewards, communication materials), and the costs of data curation and verification. Costs were estimated separately for the establishment phase (2019–2020), during which training and system development took place, and the operational phase (2021–2024), representing routine program implementation and maintenance. All costs were converted to USD using average annual conversion rates to the Indonesian Rupiah, and converted into annual costs per hectare per year, by using the total monitored area of the ANJ estates (144,611 ha).

PENDAKI costs were compared to published estimates for other biodiversity monitoring methods identified through searches of scientific and grey literature. Published wildlife monitoring costs are uncommon, and if they are it is often not clear which costs are included in costs estimates or not. Our cost comparison is not meant to be exhaustive but rather indicative.

### Ethics statement

This study used wildlife observation data collected by volunteer staff of ANJ through the PENDAKI program, which formed part of ANJ’s Responsible Development Initiative, together with data obtained through semi-structured interviews and focus group discussions conducted to evaluate program implementation. Participation in both the biodiversity monitoring program and the interviews and focus group discussions was entirely voluntary. Written informed consent was documented listing all participating employees, countersigned by the respective Project Manager and Director in charge. The purpose of the program, including identifying wildlife and analysing observational data, was clearly communicated to all potential participants. The participating estates informed company staff weekly about the program and the opportunity to participate. Interview and focus group responses were anonymised before analysis, and no individual participants are identified in this paper. No minors participated in this study. In accordance with Indonesian regulations, ethics committee approval is not required for citizen science programs involving adult company staff and therefore was not sought. All necessary permissions required in Indonesia for conducting this program were obtained.

## Results

### Participant perceptions of PENDAKI

Interviews conducted in 2022 provided insights into participants’ perceptions of the PENDAKI program. All interviewees stated that they had not previously encountered a program like PENDAKI in other companies where they had worked. When asked about the purpose of the PENDAKI, 41% described it as a method for recording species observations, 36% of respondents described the program as one which uses citizen science to monitor species, and 28% of respondents viewed it as a means of identifying species.

Eighty-two percent of respondents expressed an interest in seeing wildlife before joining PENDAKI, while 87% stated that since participating in PENDAKI their interest had increased. Respondents also stated that they had learned the name of species (12% of respondents), that they now knew which species are threatened or protected (10% of respondents), and they had gained a better understanding of the importance of protecting species and the environment (74% of respondents).

Participants identified several practical challenges in making a wildlife observation. Forty-six percent experienced challenges in taking a photo of sufficient quality for species identification, and 38% reported difficulties in species identification. Respondents from all subsidiaries referred to the PENDAKI WhatsApp groups as a means of support. The Conservation Team was frequently mentioned as providing support with species identification.

The focus group discussions held in June 2024 broadly reflected the themes from the 2022 interviews were still valid. Long-term participants described increased confidence in species identification and provided examples, including a general manager’s house cleaner who could now identify different primate species seen at the edge of the forest.

Participants also noted that levels of participation varied across the subsidiaries, citing various contributing factors such as low literacy rates in PPM and PMP in Southwest Papua. The role of the Conservation Team in motivating workers was again raised as being important. Participants discussed the reward system extensively, suggesting several refinements, although they generally indicated satisfaction with the existing reward system.

Generally, participants were satisfied with the smartphone app but identified room for improvement. This included ability to identify the 25 target species, intermittent location connectivity, and the ability to upload video and audio. During the June 2024 estate visits, RD and EM observed that the Conservation Staff had increased their species identification skills, including more frequent use of scientific names and greater familiarity with bird calls.

## Participation

We estimated that at least 3,950 individuals contributed wildlife observations between September 2019 and June 2024, although some observers may have been recorded under different names, not all contributors participated regularly, and some were no longer employed by the company at the time of analysis. Based on the ANJ workforce of 9,943 employees ANJ and its subsidiaries in 2023, this represents approximately 39% of the workforce. This estimate is likely to include employees who submitted only one or a small number of observations.

Approximately 5% of observers collected 75% of all the observations. These frequent contributors were primarily members of the Conservation Team, whose routine duties included wildlife patrols. Most other employees contributed observations only occasionally. In addition to company employees, PENDAKI also received observations from visitors to the estates, including government officials, journalists and consultants. These external contributors accounted for approximately 1.5% of all observations.

While participation in PENDAKI was voluntary for most employees, members of the Conservation Teams and senior estate management had formal responsibilities associated with program implementation and biodiversity management.

### Wildlife records and analysis

#### Data volumes, taxonomic accuracy and species groups.

Between September 2019 and June 2024, observers collected 148,286 PENDAKI wildlife records for the seven estates (Table 1). The total of correctly identified species was 699 of fauna and 186 of flora; we were unable to verify the 21 observations of fungi as we lacked the taxonomic expertise.

**Table 1:** Number of PENDAKI records across company estates. Records are differentiated by location, and were collected between September 2019 and June 2024.

Name of Estate	Key crop	Total number of PENDAKI records	Nr of records in oil palm	Nr of records in forests	Nr of records in infrastructure	Nr of records in ponds and canals
**ANJA**	Oil palm	15,393	1,758	100	2,065	11,138
**ANJAP**	Sago	12,461	4,852	1,470	1,888	1,711
**ANJAS**	Oil palm	19,406	8,768	4,804	3,638	1,783
**KAL**	Oil palm	25,575	5,787	15,451	656	1,275
**PMP**	Oil palm	14,166	11,349	1,112	1,543	82
**PPM**	Oil palm	17,042	12,928	1,649	2,022	128
**SMM**	Oil palm	44,243	20,405	7,127	2,654	13,987
**TOTAL**		**148,286**	**65,847**	**31,713**	**14,466**	**30,104**

External data reviewers classified 69% of the observations to be taxonomically Likely Correct. In addition to species reviewed as “Likely Correct” the species that were “Not Identified at Species Level” (9.2% of total observations) and “Correct ID but wrong species name” (3.8% of total observations) could also be considered correct identifications but with insufficient taxonomic precision. Overall, 12.2% of species were incorrectly identified, while 2% of the species records concerned species that specialists considered to be very difficult to identify in the field by non-specialists.

The 6.6% of species records that were “Unlikely but Possibly Correct” could be range extensions. Some 20 species records proved to be such verified range extensions; these occurred especially in Papua and on Belitung Island which remain relatively poorly surveyed. Examples included, good photographic records of Papuan Spinetail (*Mearnsia novaeguineae*) and Pied Heron (*Egretta picata*), the only confirmed sightings from Papua’s Bird’s Head region, and the description of a new mammal genus on the basis of locally taken photographs [25].

Taxonomically, most of the reliable records concerned sightings of birds and mammals, with much lower numbers for reptiles, amphibians, fish, insects and other species groups (S4 Table). The three most recorded bird species were White-throated Kingfisher (*Halcyon smyrnensis*, Sumatra and Kalimantan), Black-winged Kite (*Elanus caeruleus*, Sumatra and Kalimantan)*,* and Black-capped Lory (*Lorius lory*, Papua)*.* The three most recorded mammal species were Long-tailed Macaque (*Macaca fascicularis*, Sumatra, Belitung and Kalimantan), Large Treeshrew *(Tupaia tana*, Sumatra and Kalimantan), and Pig-tailed Macaque (*Macaca nemestrina*, Sumatra and Kalimantan). For reptiles the three most recorded species are Common Water Monitor (V*aranus salvator*, Sumatra, Belitung and Kalimantan), Reticulated Python (*Malayopython reticulatus*, Sumatra, Belitung and Kalimantan)*,* and Equatorial Spitting Cobra (*Naja sumatrana*, Sumatra, Belitung and Kalimantan).

Species records included many species of conservation concern. For example, there are 1,028 records of Bornean Orangutan, of which 829 were in forest areas and 133 in oil palm blocks. Other species that are Critically Endangered on the IUCN Red List of Threatened Species [26] included Pangolin (*Manis javanica*), Helmeted Hornbill (*Rhinoplax vigil*), and Black-spotted Cuscus (*Spilocuscus rufoniger*). Monitoring of these species is especially important, because under the Standards and Criteria of the Roundtable on Sustainable Palm Oil, the monitoring and maintenance of these species of high conservation concern is mandatory. These species are also protected by Indonesian law and can therefore not legally be harmed.

Observers collected most records (44.4%) in oil palm areas, followed by forest (21.4%), ponds and waterways (20.3%) and infrastructure (9.8%), with the remainder coming from sago areas, or unknown or unidentified locations (Table 1). The high proportion of records from oil palm areas is notable, as biodiversity surveys in plantations typically focus on species-rich forests rather than the plantations themselves, resulting in a call for greater consideration of landscape-level ecological dynamics [10].

### Examples of species occupancy to understand landscape use

**Fig 2:**
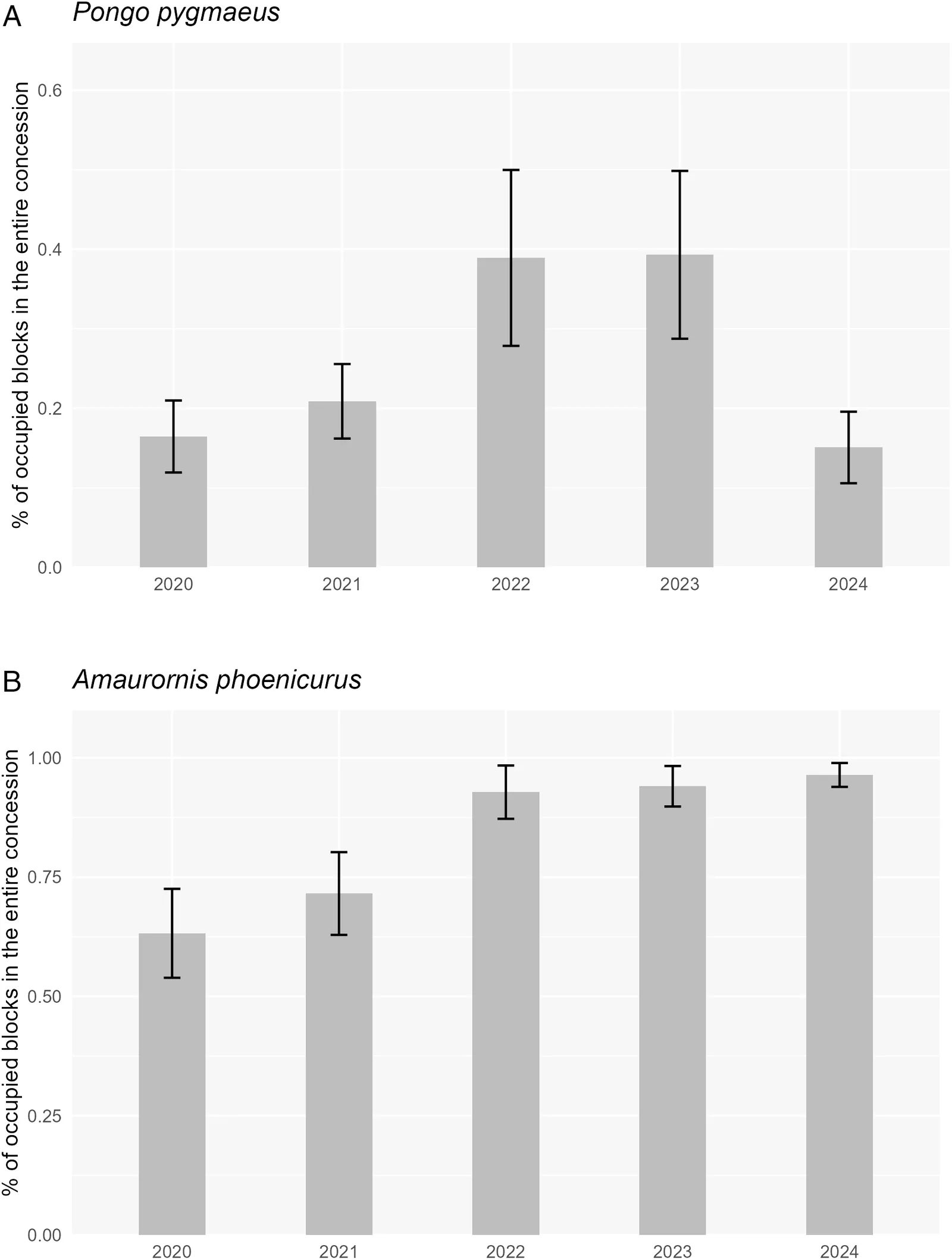
Mean percentage of blocks occupied by two example species. Fig 2A shows orangutan occupancy and Fig 2B shows the White-breasted Waterhen occupancy for the KAL estate in Ketapang, West Kalimantan.

Occupancy estimates for Orangutan and White-breasted Waterhen are presented as examples of species-specific patterns of landscape use derived from the PENDAKI dataset. Orangutans are known to survive in fragmented forest landscapes [25,26], but we know little about the spatial use of these landscapes and how plantation management could be adapted to maximize population viability. Fig 2A shows the variation between years in mean orangutan occupancy for all survey blocks in the KAL estate in West Kalimantan [for detail on methods see 16]. Orangutan occupancy in the KAL estate shows significant variation over the past 5 years, but we don’t know yet whether this truly reflects variation in orangutan abundance or behaviour, for example orangutans using oil palm areas more frequently when food was scarce. While orangutan occupancy is highest in the forest set-asides part of the KAL estate, another species, the White-breasted Waterhen has a much higher average occupancy in the planted oil palm areas (around 0.8) compared to orangutans (around 0.25) (Fig 2B).

**Fig 3:**
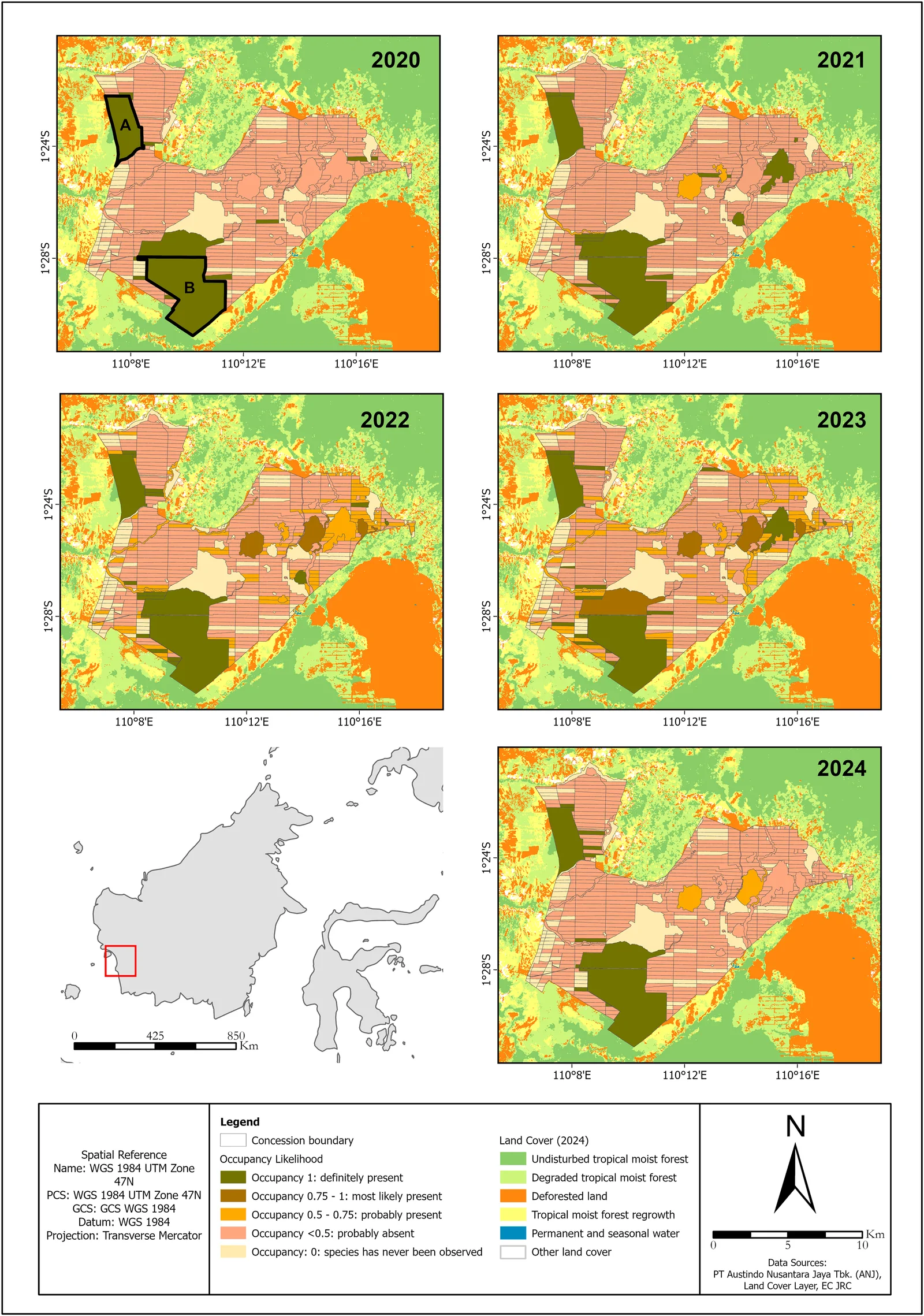
An example of the use of occupancy statistics derived from PENDAKI. The map shows occupancy values for orangutans in the PT KAL concession, in Ketapang, West Kalimantan, Indonesia. Maps are for 2020, 2021, 2022, 2023, and 2024. The letters “A” and “B” indicate breeding populations of orangutans, i.e., females are present, whereas other areas only have roaming male orangutans.

Fig 3 shows the temporal and spatial variation in orangutan occupancy for different survey blocks (forest and non-forest) in ANJ’s oil palm plantation in Kalimantan. The two large High Conservation Value forest set-asides (marked A and B in Fig 3) are areas with permanent breeding populations. Female orangutans are generally reluctant to leave forest areas, unlike male orangutans which are known to disperse through oil palm [25–27]. Monitoring orangutans in oil palm areas is very difficult because individuals are highly elusive. Consistently reported chance encounters through citizen science, however, provide a good understanding of which parts of oil palm estates are especially important for orangutan dispersal.

### Implementation costs

Implementation costs were dominated by upfront costs for technical and statistical support during the pilot phase and the development of the PENDAKI Champion application (Table 2). The annual running costs, in addition to basic conservation staff salaries, were primarily associated with rewards and internal and external reporting on PENDAKI. Annual running costs, additional to basic conservation staff salaries, were approximately USD 46,000 in the initial 2-year start-up phase and USD 20,000 thereafter. Based on ANJ’s total landbank, this equates to biodiversity monitoring costs of USD 0.32/ha (start-up) or USD 0.14/ha (long-term).

**Table 2:** Estimated annual running costs of PENDAKI during the initial 2-years pilot and the long-term implementation phase.

Cost item	Annual costs (USD)
**Pilot phase (2 years)**	
External support (training, data verification and analysis, R training reporting)	24,000
Application and dashboard development	60,000
Running costs (awards, logistics, internal training and dissemination)	8,000
**Average annual costs pilot phase**	**46,000**
**Long-term implementation (annual)**	
External support (training, data verification and analysis, R training reporting)	6,000
Application and dashboard maintenance	10,000
Running costs (awards, logistics, internal training and dissemination)	4,000
**Average annual costs long term**	**20,000**

## Discussion

### PENDAKI in the context of citizen-science monitoring frameworks

To place the PENDAKI program in context, it is useful to examine how this approach relates to the broader range of citizen science-based biodiversity monitoring frameworks described in the scientific literature. Citizen science biodiversity monitoring programs differ widely in their objectives, degree of participant involvement, and sampling design. Several frameworks have been proposed to classify these approaches. One widely used typology distinguishes between contributory, collaborative, and co-created projects, depending on whether scientists primarily design the project and volunteers mainly contribute observations, or whether participants are more actively involved in study design, interpretation, and analysis [28]. Another classification focuses on the structure of data collection, distinguishing between structured monitoring with standardized protocols, semi-structured surveys with partial guidance but flexible sampling effort, and opportunistic recording of observations made during routine activities [29,30].

Within these frameworks, the PENDAKI system most closely resembles opportunistic biodiversity monitoring, in which participants record wildlife encountered during their daily activities rather than through fixed survey protocols. Such approaches have become increasingly common in large-scale biodiversity observation networks because they can generate extensive spatial and temporal datasets that would otherwise be prohibitively costly to collect through traditional survey designs [29,31]. When analysed with appropriate statistical methods, including occupancy modelling, opportunistic observations can provide robust insights into species distribution and trends despite variation in observer effort [32,33].

At the same time, PENDAKI differs from many well-known citizen science initiatives in an important respect. Participation occurs within a corporate workforce embedded in the landscape being monitored, rather than among members of the general public or recreational naturalists. In this sense, the system combines elements of contributory citizen science with aspects of community-based monitoring, where observers encounter wildlife through their everyday activities in the landscapes they inhabit or manage. This organizational setting shapes both the scale and consistency of participation, as thousands of workers are present across plantation landscapes on a daily basis.

This hybrid model has several implications. First, it provides a mechanism for monitoring across entire estates over extended time periods without dedicated survey teams or repeated field campaigns. Second, the routine presence and mobility of workers across production landscapes, generates observation coverage that would be difficult and costly to achieve through conventional ecological surveys alone. While citizen science has been widely applied in biodiversity monitoring, corporate workforce-based monitoring systems have rarely been described. For example, a 2016 review of 162 Fortune 500 and Fortune Global 500 companies identified only one company that mentioned the use of citizen science [34]. Viewed within these citizen-science typologies, PENDAKI aligns most closely with opportunistic biodiversity monitoring in a corporate workforce-based system implemented in agricultural production landscapes.

### Corporate commitment

The interview findings suggest that visible support from senior management, together with clearly defined responsibilities for conservation and estate managers, was an important factor in the implementation of PENDAKI. Participants frequently identified leadership support as helping to establish PENDAKI during its early implementation, after which citizen science became the company’s primary approach to biodiversity monitoring. Following implementation, PENDAKI became integrated into the company’s biodiversity monitoring program and was recognised through a number of external awards and acknowledgements. This included the Roundtable on Sustainable Palm Oil (RSPO) Excellence Award for Outstanding Achievement in November 2022, followed in July 2024 by the Sustainable Marketing Excellence Award for Local Biodiversity Conservation PENDAKI [35,36]. In addition, the West Kalimantan Natural Resources Conservation Agency (*Balai Konservasi Sumber Daya Alam Kalimantan Barat – BKSDA*) formally recognized PENDAKI’s contribution to biodiversity monitoring in 2024.

Sustained engagement from both participants and management is essential to ensure the long-term success and impact of the PENDAKI system. This support now faces uncertainty, as ANJ was acquired by a new owner in May 2025, and it is unclear whether PENDAKI will continue to receive organisational support. In March 2026, the PENDAKI system was still generating wildlife observations (>189,000) under the new ownership although monthly observations counts were much reduced.

### Participation and buy-in

Data are the predominant focus of much of the published citizen science literature, with much less analysis on the factors that influence and sustain people’s participation [37]. Sustained participation by observers is widely recognised as a prerequisite for the long-term functioning of citizen science programs. As PENDAKI evolved it became clear that the system was as much about people as it was about data [38]. Similar to others [see 39,40], interview responses suggested that participation increased participants’ confidence in identifying wildlife and discussing biodiversity in the landscapes where they worked. Several participants commented that, although they had long noticed wildlife, PENDAKI provided their first opportunity to learn species identities and contribute their observations. The program also encouraged interaction among staff from different roles through a shared focus on biodiversity monitoring. PENDAKI broadened participation in biodiversity monitoring beyond conservation staff alone.

PENDAKI appeared to empower the conservation staff. The program expanded monitoring beyond conservation set asides by incorporating observations from across the entire estate. PENDAKI enabled the conservation staff to improve their species identification skills and readily identify new species for the estate. This acted as an early warning of any issues related to species, and increased engagement with the workforce.

Although participation was widespread, data collection remained highly skewed, with approximately 5% of observers contributing around 75% of all records. This pattern resembles many other citizen science programs, where a relatively small group of highly active participants contributes a disproportionate share of observations.

### Implementation capacity

The PENDAKI program was implemented largely by ANJ personnel. External support (RD and EM) was initially provided for program design, technical training and independent verification. The company was responsible for program deployment, internal training, incentive system, app and database development, and external communication. Statistical analysis was the main aspect of the program for which external expertise continued to be required [16]. This suggests that, once established, the operational requirements of the program can be integrated into existing company structures, with relatively limited ongoing reliance on external expertise.

### The value of citizen science in adaptive wildlife management

One of the greatest challenges in wildlife conservation is the measurement of impact from conservation investments on wildlife [41]. Reliably surveying wildlife populations requires large data volumes, which are expensive, time consuming to collect and difficult to interpret. The call for strengthening the evidence base for conservation [42] is challenged by the time and costs to accumulate sufficiently large datasets [43]. Furthermore, the scientific expertise that is required to analyse wildlife data often creates a distance between practitioners who are implementing conservation and data experts who analyse them [44]. This is especially the case in conservation programs implemented by rural communities or companies that have limited scientific wildlife monitoring expertise.

Our findings suggest that the PENDAKI approach can help address several practical constraints associated with biodiversity monitoring. PENDAKI has generated sufficient data to estimate spatial and temporal variations in species occupancy [16,45], while requiring relatively modest implementation costs and limited ongoing external technical support. Because observers are not taxonomic specialists, identification errors occur. Twelve percent of observations were incorrectly identified. Arguably this error rate should not affect project goals if the primary conservation target species (e.g., orangutans or gibbons) are always accurately identified. Citizen science can therefore fulfil particular but not all monitoring objectives. This is reflected in a recent review [46] that found that of the 365 indicators of the Kunming–Montreal Global Biodiversity Framework (GBF) monitoring framework, 110 (30%) can involve citizen science-based monitoring programs and 185 (51%) could benefit from citizen involvement in data collection, while 180 (49%) require scientists and governmental statistical organizations. Within these constraints, PENDAKI generates near real-time information on readily identifiable wildlife species across seven estates, including several in remote locations. These data can support ongoing monitoring of focal species and may assist managers in identifying emerging conservation issues.

### Cost comparison

Our literature review of biodiversity monitoring costs unsurprisingly showed much variation. A five-year survey effort of a 60,000 ha property in Australia using traditional methods (specialist surveys, animal traps etc.) resulted in 21,000 individual vertebrate records of 216 species for a total cost of about AUS$ 1 million (ca. USD 900,000 in 2009) [47]. This monitoring investment, equivalent to USD 15/ha, was considered insufficient for detecting species populations trends [47]. An alternative monitoring approach using ca. 10 camera traps over 2 years in an area <50 ha in the Netherlands resulted in 47,597 automatically identified species observations at a cost of EUR 66,170/y, equivalent to ca. USD 1,300/ha. Community-based monitoring tends to be much cheaper. A review of 52 community-based monitoring projects of terrestrial game fauna in the Amazon indicated average annual cost of USD 0.24/ha, while taxonomic surveys, also in the Amazon, indicated costs of USD 0.19/ha [48]. A study combining acoustic surveys with citizen science in Colombian oil palm indicated annual survey costs of less than US 3/ha [9]. We found several studies on the relative cost-effectiveness of eDNA-based biodiversity sampling [49,50], but these did not provide useable data for estimating annual and per hectare monitoring costs. It appears from this brief overview that the costs of PENDAKI are in line with other community-based monitoring methods, but probably cheaper by one or more orders of magnitude than structured survey methods that rely on more expensive technology.

The cost analysis suggests that workforce-based citizen science-based systems can provide relatively inexpensive approaches to biodiversity monitoring. In PENDAKI, implementation costs remained low because observations were contributed by existing staff, while external technical input was largely limited to statistical analysis and independent verification. The long-term annual running costs of USD 0.14/ha are substantially lower than traditional wildlife survey methods, and are similar to those reported for tropical community-based monitoring projects (USD 0.24/ha/y) [51].

## Conclusion

Our case study suggests that a corporate workforce can make a substantial contribution to long-term biodiversity monitoring through structured citizen science. The PENDAKI program generated large volumes of verified species observations that supported species inventories and occupancy analyses at relatively low implementation costs. This approach provides valuable insights into how wildlife uses plantation landscapes and how companies can help sustain it. This approach complements conventional ecological surveys and may provide a practical model for biodiversity monitoring in other large production landscapes.

## Supporting information

S1 Table Total number of respondents based on location and gender. (DOCX)

S2 File Supporting Methods. Methods used during the 2022 interviews.
(DOCX)

S3 Table Accuracy assessment drop-down options. (DOCX)

S4 Table Number of PENDAKI records per taxonomic class, and most frequently recorded taxonomic families. (DOCX)
