# Supplementary material for: Citizen science-based biodiversity monitoring in oil palm plantations
Source: PLoS One. 2026 Aug 25;21(8):e0318046. doi: 10.1371/journal.pone.0318046 (PMC13505915; doi:10.1371/journal.pone.0318046)
Supplement: Supplementary file 1 [file pone.0318046.s001.docx]

Table S1. Total number of respondents based on location and gender

| Location | Respondents | Male | Female |
| --- | --- | --- | --- |
| Head Office Jakarta | 6 | 4 | 2 |
| PMP | 6 | 5 | 1 |
| PPM | 5 | 4 | 1 |
| KAL | 6 | 4 | 2 |
| ANJAS | 5 | 5 | 0 |
| ANJA | 6 | 4 | 2 |
| SMM | 5 | 4 | 1 |
| Totals | 39 | 30 | 9 |
