# Supplementary material for: Citizen science-based biodiversity monitoring in oil palm plantations
Source: PLoS One. 2026 Aug 25;21(8):e0318046. doi: 10.1371/journal.pone.0318046 (PMC13505915; doi:10.1371/journal.pone.0318046)
Supplement: Supplementary file 2 [file pone.0318046.s002.docx]

**S2 Supporting Methods. Methods used during the 2022 interviews.**

The interviews were conducted between 9th and 27th June 2022, with the bulk of the interviews taking place between the 9^th^ and 16^th^ of June. Thirty-nine interviews were conducted, with between 5 and 6 interviewees preselected by ANJ staff from each of the 6 subsidiaries and head office in Jakarta (Table S1).

The interviews were conducted by video call (Zoom) for the subsidiaries and in person for head office. The interview comprised 13 questions (see below) which took about 20 minutes to run through. The interviews were conducted mostly on a one-to-one basis, notes were taken, and no audio recordings were made. In some instances, the on-site company conservation staff assisted if questions were not understood by the respondent. The timeframe for the interviews was kept to the afternoons to ensure that respondents could join the interview when they had just finished their work. The interviews were mostly conducted in Indonesian, with some of the interviews at head office conducted in English. The list of questions was discussed in advance with the Conservation Manager. The notes taken during the interview were reviewed and entered into an Excel spreadsheet in order to perform some basic statistics to identify trends in responses. The interview questions were as follows:

1. What are your daily work activities and where?
2. Describe the Pendaki Program.
3. Why do you participate in Pendaki?
4. If you are interested in wildlife, have you always been interested.
5. Has your level of interest in wildlife increased or changed since you started participating in Pendaki?
6. What type of species have you seen and what is the most memorable/unique?
7. Tell me how you do an observation.
8. What challenges do you have when you make an observation?
9. How could Pendaki be improved?
10. What do you think about an app based Pendaki?
11. How many observations do you do in a month, and have you received an award?
12. Do you talk to your family and friends about Pendaki and the wildlife that you see?
13. Have you ever heard of a program like Pendaki in another company?
