# Supplementary material for: Citizen science-based biodiversity monitoring in oil palm plantations
Source: PLoS One. 2026 Aug 25;21(8):e0318046. doi: 10.1371/journal.pone.0318046 (PMC13505915; doi:10.1371/journal.pone.0318046)
Supplement: Supplementary file 3 [file pone.0318046.s003.docx]

S3 Table. Accuracy assessment drop-down options. These are the choices in the species verification process to determine the likely accuracy of a species identification.

| Drop-down menu options | Description |
| --- | --- |
| Likely correct | The species is verified to occur in the area and ecosystem where it was recorded. |
| Unlikely but possibly correct | The species is either very rare in or thought to be absent from the area where it was recorded (e.g., a rare migrant), but its presence cannot be excluded. |
| Probably incorrect | The typical ecosystem of this species (e.g., montane forest) does not occur in the plantation area. |
| Most likely incorrect | The species has never been recorded in the broad vicinity of the plantation or on the island where it was reported from. |
| Correct ID but wrong species name | The species was likely correctly identified but the wrong species name was given (or the species name has recently been updated in a taxonomic revision). |
| Very difficult to identify | The species looks very similar to a closely related species and can only be reliably identified to species level by a species expert or when it is caught and closely examined. |
| Not identified at species level | The record does not contain a species name but only a general description (e.g., owl or *Harpectes* sp.). |
