# Supplementary material for: Citizen science-based biodiversity monitoring in oil palm plantations
Source: PLoS One. 2026 Aug 25;21(8):e0318046. doi: 10.1371/journal.pone.0318046 (PMC13505915; doi:10.1371/journal.pone.0318046)
Supplement: Supplementary file 4 [file pone.0318046.s004.docx]

S4 Table. Number of PENDAKI records per taxonomic class, and most frequently recorded taxonomic families

| Species group | Taxonomic class | Number of records per class | Three most frequently recorded species |
| --- | --- | --- | --- |
| Birds | Aves | 91,211 | *Halcyon smyrnensis, Elanus caeruleus, Lorius lory* |
| Mammals | Mammalia | 28,086 | *Macaca fascicularis, Tupaia tana, Macaca nemestrina* |
| Snakes, turtles and lizards | Reptilia | 20,032 | *Varanus salvator, Malayopython reticulatus, Naja sumatrana* |
| Dicot plants | Magnoliopsida | 4,025 | *Nepenthes ampullaria, N. bicalcarata, N. rafflesiana* |
| Insects | Insecta | 2,025 |  |
| Lilies, orchids, grasses | Liliopsida | 1,085 |  |
| Fishes | Actinopterygii | 1,276 |  |
| Spiders and scorpions | Arachnida | 135 |  |
| Frogs | Amphibia | 150 |  |
| Fungi and slime moulds | Agaricomycetes and Myxomycetes | 34 |  |
| Centipedes | Chilopoda | 94 |  |
| Snails | Gastropoda | 12 |  |
| Horsetails | Equisetopsida | 3 |  |
| Millipedes | Diplopoda | 26 |  |
| Others and unknown |  | 90 |  |
