# Supplementary material for: Citizen science-based biodiversity monitoring in oil palm plantations
Source: PLoS One. 2026 Aug 25;21(8):e0318046. doi: 10.1371/journal.pone.0318046 (PMC13505915; doi:10.1371/journal.pone.0318046)
Supplement: Supplementary file 5 [file pone.0318046.s006.docx]

We thank the editor and reviewer for providing feedback on our initial manuscript. We agree that the original structure and language required improvement, and that we needed to provide a lot more detail on the statistical modelling. The latter we have done by submitting a separate manuscript which we hereby attach.

Comment 1. The authors have produced a large dataset, seemingly for relatively low cost. It potentially has substantial value. However, the manuscript in its current form is difficult to follow and lacks a focus and clear message. What is the manuscript specifically trying to show, or what question is being answered? Is it to demonstrate this approach to citizen science data collection? Is it about orangutans, for which there an example of occupancy modelling? Is it about occupancy analysis more broadly? Is it the Living Plantation Index? There seems to be a mixture of all these and more. In many ways this reads like a project report for a funder rather than a planned scientific paper.

Response 1. We very much appreciate the constructive guidance from the editor and the reviewer and apologize for presenting a rather poorly structured manuscript that tried to present too many different aspects of our citizen science pilot study. Based on your feedback, we have significantly restructured the original manuscript and present a shorter and clearer revision. We have simultaneously submitted an additional manuscript to PLOS ONE that describes the occupancy analysis in much more detail, showing that the dataset is indeed robust. Incorporating that statistical analysis into the current manuscript would have made it too long, and probably also end up trying to target two distinct audiences, those interested in statistical modeling and those interested in practical solutions to wildlife monitoring in agricultural context.

We hope that the current manuscript is much more clearly structured around the four research questions at the end of the Introduction, with the sequence of these questions now being repeated in the Methods, Results and Discussion. We hope that with these changes, and a companion paper on the statistical models, we offer readers of PLOS ONE a much clearer and comprehensive overview of the potential value of citizen science-based wildlife monitoring in agricultural landscapes.

Comment 2. The occupancy modelling in particular seems out of place, yet also central to some messages. It is difficult to evaluate the Bayesian occupancy modeling since the details are absent and appear to be for a separate manuscript. Lines 127-128 – It is stated, “Detailed description of statistical methods and modelling assumptions for developing occupancy statistics from the PENDAKI data will be published elsewhere.” Also see lines 189-190. Why isn’t this part of the current manuscript?

Response 2. We considered incorporating the occupancy modelling into the manuscript, but this is a much more technical text that focuses on the specifics of the statistical models. Our current manuscript targets an audience of conservation practitioners working in palm oil or other agricultural contexts or for conservation NGOs. The second manuscript has a more scientific, and statistically trained target audience. We think it is better to present these two studies separately. We attach the second manuscript to this submission so that the editor and reviewer can access the information. We have also removed most references to the statistical analyses from the current manuscript as these are now addressed in the second manuscript.

Comment 3. The reviewer who would review the manuscript also had concerns about how it was written and the lack of clarity on the message. My suggestion is to decide exactly what the main message and goal of this manuscript is, remove the parts that are not relevant, and add anything that is absolutely necessary for a reviewer to understand and evaluate what you did. This can all potentially be fixed, but the authors will need to substantially rewrite what they have done. This may also result in the need for further review.

Response 3. Thank you for your guidance on this. We have significantly restructured the manuscript, with the 4 research questions at the end of the Introduction now sequentially addressed in the Methods, Results, and Discussion. As already mentioned, we have deleted the statistical modelling part and submitted a separate manuscript that describes this in much more detail.


Comment 4. The [PLOS Data policy](http://track.editorialmanager.com/CL0/http:%2F%2Fwww.plosone.org%2Fstatic%2Fpolicies.action%23sharing/1/010f01972edb02b3-5fdf0ba4-6091-43c2-a4ef-9967da6bad8f-000000/TwzeBOLVT8UAPy0ICk4irAljIJI05cpSxAQRgrQ0W6Y=213) requires authors to make all data underlying the findings described in their manuscript fully available without restriction, with rare exception (please refer to the Data Availability Statement in the manuscript PDF file). The data should be provided as part of the manuscript or its supporting information, or deposited to a public repository. For example, in addition to summary statistics, the data points behind means, medians and variance measures should be available. If there are restrictions on publicly sharing data—e.g. participant privacy or use of data from a third party—those must be specified.

Response 4. We have made the raw data available at Zenodo pending final acceptance of the current manuscript: 10.5281/zenodo.15845290.

Reviewer #1: This is an interesting and important study - however I found that the presentation style needed significant improvement. The English language is correct, but the style is inappropriate for a journal paper. There is too much use of the passive tense, too many long sentences, a lot of relatively unimportant text is included and there is an absence of clear and direct statements of methods and results. Too much contextual text and a lot of text which only communicates options

Response 5. Thank you Dr Sayer; we greatly appreciate your feedback. We agree with you and the editor that the manuscript required significant rewriting and restructuring, which we have done. We have also removed information not directly relevant to the 4 research questions, and developed a separate manuscript on the occupancy modelling.
