# Supplementary material for: Citizen science-based biodiversity monitoring in oil palm plantations
Source: PLoS One. 2026 Aug 25;21(8):e0318046. doi: 10.1371/journal.pone.0318046 (PMC13505915; doi:10.1371/journal.pone.0318046)
Supplement: Supplementary file 6 [file pone.0318046.s007.docx]

**Editor comments**

Comment 1. … the paper needs substantial revision before it’s ready for PLOS ONE. The biggest gap is methodological transparency. The manuscript notes that the technical details of the Bayesian occupancy models will be published elsewhere. That’s not sufficient for a standalone article. Readers need the full model structure, priors, treatment of detection probability, how uneven sampling effort was handled, spatial autocorrelation considerations, convergence diagnostics, and software specifics. Without these, the work isn’t reproducible and the robustness of the findings can’t be fairly assessed.

Response 1. Our target audience for the current paper is practitioners in the plantation industry. For that reason, we have kept the language in the manuscript non-technical and decided to publish the statistical model separately for readers who are interested in the statistical details. This paper has now been published in PLOS ONE and we refer to it in the current manuscript: <https://journals.plos.org/plosone/article?id=10.1371/journal.pone.0328960>. This paper is open access and therefore readers who are interested in the full model structure, use of priors, the use of detection probability to correct for sampling effort, and uneven spatial sampling and others, can refer to this online paper. Incorporating these model details into the current manuscript would make it unnecessarily lengthy and less suitable for a practitioner’s audience.

We hope that the editor agrees with us that there is value in providing practitioners in the palm oil industry with a broad overview of the use of citizen science-type approaches in wildlife monitoring in this highly scrutinized industry. We believe that the current manuscript meets PLOS ONE objectives of having a strong methodology in its detailed description of how the citizen science methods were implemented in the field, what the key elements were to its success, and what the practicalities are of using it in a palm oil context (uptake, cost, analytical output options, perceptions among work force).

Comment 2. A related issue is sampling bias. Because observations are opportunistic and effort isn’t standardized, spatial and temporal biases are likely. Workers will tend to record species near roads, infrastructure, and frequently visited blocks; shifting reporting intensity over time could mimic trends. While occupancy models address imperfect detection, it’s not clear that observer heterogeneity, habitat-specific detectability, or variation in effort were adequately modeled. The paper would benefit from a sharper discussion here, and ideally a quantitative assessment of how these biases were diagnosed and mitigated.

Response 2. The reviewer is right in saying that opportunistic data suffer from spatial and temporal biases; therefore, a statistical technique (occupancy modeling) was chosen that enables to consider these difficulties.

Occupancy models were originally designed to estimate true occupancy by correcting for differences in detection probability. But they can also be used as a tool to correct for unknown sampling effort. We explain in the separate paper mentioned above that this is a well-established model for analysis of citizen science data; see references in that paper). In short, the idea is that a higher sampling effort leads to a higher detection probability of a species within a block, provided the block is being occupied (production blocks in plantations were our unit of analysis). Blocks near roads and infrastructure are visited more frequently and less accessible blocks get fewer visits, which will lead to differences in reporting rate of a species between blocks. The model corrects for differences in the number of visits while estimating the occupancy probability of blocks. The same applies to changes in sampling effort over time. The model also distinguishes between the number of reported species – a longer list is often associated with higher sampling effort – , between observers to account for observer heterogeneity and between habitats to account for habitat-specific detection. In addition, obvious risks of spatial sampling bias were addressed in the companion paper. More particular, we tested whether blocks surveyed only once in 2020-2024 may lead to biased results. The results without these blocks, however, were not different than including them.

For these details on the statistical model, we refer the reader to the companion paper in PLOS ONE. The objective of the current paper is to convince our target audience of conservation practitioners and the agricultural sector that wildlife observations involving company work forces can usefully contribute to their adaptive management requirements. In our experience, this target audience is less interested in the statistical details than in practical questions about how to set up a program like this, cost considerations, and potential outputs from data analysis.

Comment 3. The Living Plantation Index (LPI) is a creative idea, but its construction feels somewhat ad hoc. The weighting scheme that blends IUCN status, national protection, CITES listing, and range category needs either stronger theoretical grounding or evidence of robustness. A sensitivity analysis showing how alternative weights change the results would go a long way toward making the index scientifically defensible rather than merely communicative.

Response 3. This is odd, because after the first round of reviews, we had removed the Living Plantation Index from the manuscript. In the revised version, there is no reference to this, and we wonder whether the editor has indeed seen the most up-to-date version of the manuscript. Could the editor please verify that the manuscript number PONE-D-24-59415_R1 was under review.

Comment 4. Some interpretations—especially around orangutan landscape use and corridor functionality—lean beyond what occupancy alone can support. Occupancy is not abundance, and non-detection is not proof of absence. These claims should be tempered to match the inferential limits of the modeling framework.

Response 4. Thank you for the comment.

We think that the make the distinction between occupancy and abundance quite clearly in the text. For example, in lines 337-340, we write “Orangutan occupancy in the KAL estate shows significant variation over the past 5 years, but we don’t know yet whether this truly reflects variation in orangutan abundance or behaviour, for example orangutans using oil palm areas more frequently when food was scarce.”

But we do not agree with the suggestion of the reviewer that we have only information about non-detections and not about absences of a species. Occupancy models enable to estimate the probability of real absences; these estimates are derived from non-detection records and the detection probability estimates.

Comment 5. The interview-based social assessment is a useful complement but should be read cautiously given small samples, participant pre-selection, and possible social desirability bias. Likewise, sections highlighting ESG reporting gains and corporate recognition would benefit from a more neutral, scientific tone to avoid sounding promotional.

Response 5. Thank you for the comment, and we have revised this text section to ensure a more neutral tone and more careful interpretation of the interview results. We also added a caveat in the Results section that draws attention to the small sample size and selection bias. Despite the small sample size and selection bias, we believe that the interviews provided important insights from partic

Comment 6. In sum, this is an important, large-scale attempt to integrate citizen science into corporate biodiversity management, with clear applied value and a promising analytical approach. To be publication-ready, it needs major revisions that: make the modeling fully transparent and reproducible; address sampling bias more rigorously; justify and stress-test the index; clarify potential conflicts of interest; and moderate conclusions to fit the evidence. With these improvements, the study could make a meaningful contribution to conservation monitoring and citizen science in production landscapes.

Response 6. Thank you for the supportive comments. We hope that the editor agrees that the companion paper with the statistical modelling details provides a suitable reference point for the readers to critically interpret the findings and claims in the current manuscript.

Comment 7. Reviewer #2: The work presented by the authors is truly inspiring and demonstrates an impressive integration of citizen participation in ecological monitoring. Your model of engagement and implementation provides valuable lessons for large-scale, community-driven research. The clarity of their methodological design makes this study a notable contribution to the growing field of citizen science.
That said, I would be very interested to see a more detailed discussion regarding how the authors addressed spatial and temporal biases inherent in citizen-science data collection. While occupancy models can indeed accommodate imperfect detection, they do not automatically correct for the non-random spatial distribution of effort or the temporal clustering of sampling events typical of volunteer-based monitoring. In such cases, detection probability may still be spatially autocorrelated with accessibility and human density, leading to systematic sampling. It would be good to write how you address those biases throe experiment design and data management.

Response 7. Thank you for your constructive feedback. As mentioned above, we decided to publish the statistical modeling details separately (<https://journals.plos.org/plosone/article?id=10.1371/journal.pone.0328960>).

In that study we used the same citizen science data discussed in the current manuscript.

See our Response 2 above how we dealt with the biases inherent in opportunistic data.

We understand that ideally these statistical details would have been covered in the current manuscript, but because we believe that our target audience for the current manuscript is more likely to read a less technical paper that describes the approach and its potential benefits in more general terms, leaving the statistical paper for readers who are more interested in methodological details.

Comment 8. In addition, I suggest situating the study within the broader landscape of citizen-science frameworks by discussing whether and how this work aligns with or diverges with other established models of citizen science.
Overall, this is a strong and well-executed manuscript. Addressing the two points above would further strengthen the paper and firmly position it within the wider discourse on citizen-science operational models.

Response 8.

Thank you for this helpful suggestion. We have added new text to the start of the Discussion that situates the PENDAKI program within commonly used citizen-science typologies. In particular, we note that citizen-science initiatives are often classified according to (i) the degree of participant involvement (e.g., contributory, collaborative, or co-created projects) and (ii) the structure of the monitoring design (structured, semi-structured, or opportunistic data collection). We clarify that PENDAKI most closely resembles semi-structured or opportunistic biodiversity monitoring systems in which observers record wildlife encountered during routine activities rather than through fixed survey protocols.

At the same time, we emphasize that PENDAKI differs from many citizen-science initiatives because participation occurs within a corporate workforce rather than among the general public. This creates a hybrid form of citizen science that combines elements of opportunistic biodiversity observation with aspects of workplace-based or community monitoring. We added text highlighting how this model leverages the continuous presence of estate workers across large landscapes, allowing biodiversity monitoring at spatial and temporal scales that would be difficult to achieve through conventional survey approaches. These additions help position the study within the broader citizen-science literature and clarify how the PENDAKI approach relates to existing frameworks.
