# Supplementary material for: Citizen science-based biodiversity monitoring in oil palm plantations
Source: PLoS One. 2026 Aug 25;21(8):e0318046. doi: 10.1371/journal.pone.0318046 (PMC13505915; doi:10.1371/journal.pone.0318046)
Supplement: Supplementary file 7 [file pone.0318046.s008.docx]

| Editor Comments |  |
| --- | --- |
| First thing is around the description of the occupancy modelling approach. Acknowledging published methodology separately, the current manuscript's focus for a practitioner audience, many readers will only read the present paper separately. Therefore, it would be helpful to include a brief, non-technical summary of how the analyses accounted for issues such as detection probability, variation in observer effort, and differences among habitats and observers. A concise explanation would improve accessibility without substantially increasing the technical complexity of the manuscript. | We have inserted a paragraph that summarizes the occupancy methods and assumptions in simple words (Lines 193-205), while maintaining the reference to the van Strien et al. (2025) paper for technical details. We hope that this high-level description sufficiently clarifies the modelling approach for non-technical readers. |
| Secondly around the tone, because current version of manuscript may be interpreted as promotional or advocacy-oriented lengthy awards discussion. Several statements describing the programme as a “success”, or linking it directly to improvements in the company’s environmental performance and reputation, would benefit from a more neutral scientific framing. The strength of the manuscript lies in the evidence presented, and the conclusions will be more convincing if they are expressed in a measured and objective manner. Similarly, authors can consider the discussion of awards and external recognition, to be shortened or reframed, so that it provides context rather than serving as evidence of programme effectiveness. | We thank the Editor for this helpful observation and agree that several sections of the manuscript could be interpreted as overly promotional. We have undertaken a thorough revision of the manuscript to adopt a more neutral scientific tone throughout. In particular, we have:   - removed evaluative terms such as successful, key to success, low-cost, important, critical, and empowered where these were not directly supported by the data; - separated descriptive results from their interpretation, moving management implications and broader conclusions from the Results into the Discussion where appropriate; - rewritten several Discussion sections so that interpretations are explicitly linked to interview findings or quantitative results rather than presented as general statements; - shortened and reframed the discussion of awards and external recognition so that these provide context regarding external acknowledgement rather than evidence of programme effectiveness. |
| This is around response on online submission portal, declaring conflict of interests: Instead of sayign 'no competing interests, authors may mention "Several authors are employees of ANJ, the company where PENDAKI was implemented." Since the manuscript evaluates an ANJ-developed program, it may be a perceived conflict from editorial view (not technically). | We agree and have changed the COI statement in the portal accordingly. |
| The participation results are interesting and, yes, they represent one of the strengths of the paper. At the same time, manuscript reports that 'a relatively small proportion of contributors generated the majority of observations'. Authors consider to reflect on this point in the Discussion section, or to acknowledge that long-term biodiversity monitoring may depend heavily on a relatively small group of highly engaged participants. This would provide a balanced interpretation of programme uptake and sustainability. | We agree that this is an important point. We have expanded the Discussion to acknowledge that participation was highly skewed, with approximately 5% of observers contributing around 75% of all wildlife observations. We discuss this in the context of wider citizen science literature, noting that such participation patterns are common in volunteer-based monitoring programmes, and we consider the implications for long-term programme sustainability. |
| This manuscript clearly shows that the programme can generate large quantities of useful biodiversity information, however, evidence (for programme directly improving biodiversity status or conservation outcomes) is relatively beyond the scope of the current study. Hence it can be toned down relevantly. | We agree and have revised the manuscript accordingly. We have removed or softened statements implying that PENDAKI directly improved biodiversity outcomes or conservation status. The revised manuscript now distinguishes more clearly between (i) evidence demonstrating the programme's capacity to generate biodiversity information and (ii) the potential management applications of these data, which are discussed as implications rather than demonstrated outcomes. |
| In general, please undertake a final check of language, grammar, reference consistency and data availability information prior to submission. | In response to the Editor's comments, we also reviewed the structure of the manuscript to improve the separation between Results and Discussion. Descriptions of database management and validation procedures have been relocated to the Methods where appropriate, while interpretive statements and management implications have been transferred from the Results to the Discussion. This has resulted in a more objective presentation of the findings. |
