# Supplementary material for: Citizen science-based biodiversity monitoring in oil palm plantations
Source: PLoS One. 2026 Aug 25;21(8):e0318046. doi: 10.1371/journal.pone.0318046 (PMC13505915; doi:10.1371/journal.pone.0318046)
Supplement: Peer review history

# Peer review: Decision Letter 0

**DOI:** 10.1371/journal.pone.0318046.r001

**Authors:** Clinton Jenkins

2 Jun 2025

A novel citizen science-based wildlife monitoring and management tool for oil palm plantations

PLOS ONE

Dear Dr. Meijaard,

Thank you for submitting your manuscript to PLOS ONE. After careful consideration, we feel that it has merit but does not fully meet PLOS ONE’s publication criteria as it currently stands. Therefore, we invite you to submit a revised version of the manuscript that addresses the points raised during the review process.

Please submit your revised manuscript by Jul 17 2025 11:59PM. If you will need more time than this to complete your revisions, please reply to this message or contact the journal office at plosone@plos.org. When you're ready to submit your revision, log on to https://www.editorialmanager.com/pone/ and select the 'Submissions Needing Revision' folder to locate your manuscript file.

- A rebuttal letter that responds to each point raised by the academic editor and reviewer(s). You should upload this letter as a separate file labeled 'Response to Reviewers'.
- A marked-up copy of your manuscript that highlights changes made to the original version. You should upload this as a separate file labeled 'Revised Manuscript with Track Changes'.
- An unmarked version of your revised paper without tracked changes. You should upload this as a separate file labeled 'Manuscript'.

If you would like to make changes to your financial disclosure, please include your updated statement in your cover letter. Guidelines for resubmitting your figure files are available below the reviewer comments at the end of this letter.

If applicable, we recommend that you deposit your laboratory protocols in protocols.io to enhance the reproducibility of your results. Protocols.io assigns your protocol its own identifier (DOI) so that it can be cited independently in the future. For instructions see: https://journals.plos.org/plosone/s/submission-guidelines#loc-laboratory-protocols. Additionally, PLOS ONE offers an option for publishing peer-reviewed Lab Protocol articles, which describe protocols hosted on protocols.io. Read more information on sharing protocols at https://plos.org/protocols?utm_medium=editorial-email&utm_source=authorletters&utm_campaign=protocols.

We look forward to receiving your revised manuscript.

Kind regards,

Clinton N. Jenkins, Ph.D.

Academic Editor

PLOS ONE

Journal Requirements:

When submitting your revision, we need you to address these additional requirements.

1. Please ensure that your manuscript meets PLOS ONE's style requirements, including those for file naming. The PLOS ONE style templates can be found at

https://journals.plos.org/plosone/s/file?id=wjVg/PLOSOne_formatting_sample_main_body.pdf and

https://journals.plos.org/plosone/s/file?id=ba62/PLOSOne_formatting_sample_title_authors_affiliations.pdf

2. You indicated that ethical approval was not necessary for your study. We understand that the framework for ethical oversight requirements for studies of this type may differ depending on the setting and we would appreciate some further clarification regarding your research. Could you please provide further details on why your study is exempt from the need for approval and confirmation from your institutional review board or research ethics committee (e.g., in the form of a letter or email correspondence) that ethics review was not necessary for this study? Please include a copy of the correspondence as an "Other" file.

3. Please provide additional details regarding participant consent. In the ethics statement in the Methods and online submission information, please ensure that you have specified (1) whether consent was informed and (2) what type you obtained (for instance, written or verbal, and if verbal, how it was documented and witnessed). If your study included minors, state whether you obtained consent from parents or guardians. If the need for consent was waived by the ethics committee, please include this information.

If you are reporting a retrospective study of medical records or archived samples, please ensure that you have discussed whether all data were fully anonymized before you accessed them and/or whether the IRB or ethics committee waived the requirement for informed consent. If patients provided informed written consent to have data from their medical records used in research, please include this information.

4. Please include a complete copy of PLOS’ questionnaire on inclusivity in global research in your revised manuscript. Our policy for research in this area aims to improve transparency in the reporting of research performed outside of researchers’ own country or community. The policy applies to researchers who have travelled to a different country to conduct research, research with Indigenous populations or their lands, and research on cultural artefacts. The questionnaire can also be requested at the journal’s discretion for any other submissions, even if these conditions are not met.  Please find more information on the policy and a link to download a blank copy of the questionnaire here: https://journals.plos.org/plosone/s/best-practices-in-research-reporting. Please upload a completed version of your questionnaire as Supporting Information when you resubmit your manuscript.

5. When completing the data availability statement of the submission form, you indicated that you will make your data available on acceptance. We strongly recommend all authors decide on a data sharing plan before acceptance, as the process can be lengthy and hold up publication timelines. Please note that, though access restrictions are acceptable now, your entire data will need to be made freely accessible if your manuscript is accepted for publication. This policy applies to all data except where public deposition would breach compliance with the protocol approved by your research ethics board. If you are unable to adhere to our open data policy, please kindly revise your statement to explain your reasoning and we will seek the editor's input on an exemption. Please be assured that, once you have provided your new statement, the assessment of your exemption will not hold up the peer review process.

6. Please amend the manuscript submission data (via Edit Submission) to include author Nardiyono.

7. Please amend your authorship list in your manuscript file to include author N Nardiyono.

8. We note that Figures 1 and 3 in your submission contains map images which may be copyrighted. All PLOS content is published under the Creative Commons Attribution License (CC BY 4.0), which means that the manuscript, images, and Supporting Information files will be freely available online, and any third party is permitted to access, download, copy, distribute, and use these materials in any way, even commercially, with proper attribution. For these reasons, we cannot publish previously copyrighted maps or satellite images created using proprietary data, such as Google software (Google Maps, Street View, and Earth). For more information, see our copyright guidelines: http://journals.plos.org/plosone/s/licenses-and-copyright.

We require you to either (1) present written permission from the copyright holder to publish these figures specifically under the CC BY 4.0 license, or (2) remove the figures from your submission:

1. You may seek permission from the original copyright holder of Figure 1 and 3 to publish the content specifically under the CC BY 4.0 license.

We recommend that you contact the original copyright holder with the Content Permission Form (http://journals.plos.org/plosone/s/file?id=7c09/content-permission-form.pdf) and the following text:

“I request permission for the open-access journal PLOS ONE to publish XXX under the Creative Commons Attribution License (CCAL) CC BY 4.0 (http://creativecommons.org/licenses/by/4.0/). Please be aware that this license allows unrestricted use and distribution, even commercially, by third parties. Please reply and provide explicit written permission to publish XXX under a CC BY license and complete the attached form.”

Please upload the completed Content Permission Form or other proof of granted permissions as an "Other" file with your submission.

In the figure caption of the copyrighted figure, please include the following text: “Reprinted from [ref] under a CC BY license, with permission from [name of publisher], original copyright [original copyright year].”

2. If you are unable to obtain permission from the original copyright holder to publish these figures under the CC BY 4.0 license or if the copyright holder’s requirements are incompatible with the CC BY 4.0 license, please either i) remove the figure or ii) supply a replacement figure that complies with the CC BY 4.0 license. Please check copyright information on all replacement figures and update the figure caption with source information. If applicable, please specify in the figure caption text when a figure is similar but not identical to the original image and is therefore for illustrative purposes only.

The following resources for replacing copyrighted map figures may be helpful:

USGS National Map Viewer (public domain): http://viewer.nationalmap.gov/viewer/

The Gateway to Astronaut Photography of Earth (public domain): http://eol.jsc.nasa.gov/sseop/clickmap/

Maps at the CIA (public domain): https://www.cia.gov/library/publications/the-world-factbook/index.html and https://www.cia.gov/library/publications/cia-maps-publications/index.html

NASA Earth Observatory (public domain): http://earthobservatory.nasa.gov/

Landsat: http://landsat.visibleearth.nasa.gov/

USGS EROS (Earth Resources Observatory and Science (EROS) Center) (public domain): http://eros.usgs.gov/#

Natural Earth (public domain): http://www.naturalearthdata.com/

Additional Editor Comments:

First, apologies for the long delay in reaching a decision on your manuscript. Finding reviewers is becoming increasingly difficult.

The broad topic of your manuscript, the utility of citizen science for biodiversity monitoring, is of interest in the conservation science and management community. Oil palm plantations are also of wide interest because of their massive impact on biodiversity, mainly through the transformation of former wet tropical forests.

The authors have produced a large dataset, seemingly for relatively low cost. It potentially has substantial value. However, the manuscript in its current form is difficult to follow and lacks a focus and clear message. What is the manuscript specifically trying to show, or what question is being answered? Is it to demonstrate this approach to citizen science data collection? Is it about orangutans, for which there an example of occupancy modelling? Is it about occupancy analysis more broadly? Is it the Living Plantation Index? There seems to be a mixture of all these and more. In many ways this reads like a project report for a funder rather than a planned scientific paper.

The occupancy modelling in particular seems out of place, yet also central to some messages. It is difficult to evaluate the Bayesian occupancy modeling since the details are absent and appear to be for a separate manuscript. Lines 127-128 – It is stated, “Detailed description of statistical methods and modelling assumptions for developing occupancy statistics from the PENDAKI data will be published elsewhere.” Also see lines 189-190. Why isn’t this part of the current manuscript?

The reviewer who would review the manuscript also had concerns about how it was written and the lack of clarity on the message.

My suggestion is to decide exactly what the main message and goal of this manuscript is, remove the parts that are not relevant, and add anything that is absolutely necessary for a reviewer to understand and evaluate what you did. This can all potentially be fixed, but the authors will need to substantially rewrite what they have done. This may also result in the need for further review.

[Note: HTML markup is below. Please do not edit.]

Reviewers' comments:

Reviewer's Responses to Questions

**Comments to the Author**

1. Is the manuscript technically sound, and do the data support the conclusions?

Reviewer #1: Yes

**********

2. Has the statistical analysis been performed appropriately and rigorously?

Reviewer #1: I Don't Know

**********

3. Have the authors made all data underlying the findings in their manuscript fully available?

The PLOS Data policy

Reviewer #1: Yes

**********

4. Is the manuscript presented in an intelligible fashion and written in standard English?

Reviewer #1: Yes

**********

Reviewer #1: This is an interesting and important study - however I found that the presentation style needed significant improvement. The English language is correct, but the style is inappropriate for a journal paper. There is too much use of the passive tense, too many long sentences, a lot of relatively unimportant text is included and there is an absence of clear and direct statements of methods and results. Too much contextual text and a lot of text which only communicates options

**********

what does this mean?). If published, this will include your full peer review and any attached files.

If you choose “no”, your identity will remain anonymous but your review may still be made public.

**Do you want your identity to be public for this peer review?**  For information about this choice, including consent withdrawal, please see our Privacy Policy

Reviewer #1: **Yes:**  Jeffrey Sayer

**********

[NOTE: If reviewer comments were submitted as an attachment file, they will be attached to this email and accessible via the submission site. Please log into your account, locate the manuscript record, and check for the action link "View Attachments". If this link does not appear, there are no attachment files.]

While revising your submission, please upload your figure files to the Preflight Analysis and Conversion Engine (PACE) digital diagnostic tool, https://pacev2.apexcovantage.com/. PACE helps ensure that figures meet PLOS requirements. To use PACE, you must first register as a user. Registration is free. Then, login and navigate to the UPLOAD tab, where you will find detailed instructions on how to use the tool. If you encounter any issues or have any questions when using PACE, please email PLOS at figures@plos.org. Please note that Supporting Information files do not need this step.

# Author comment: Author response to Decision Letter 1

**DOI:** 10.1371/journal.pone.0318046.r002

10 Jul 2025

Comment 1. The authors have produced a large dataset, seemingly for relatively low cost. It potentially has substantial value. However, the manuscript in its current form is difficult to follow and lacks a focus and clear message. What is the manuscript specifically trying to show, or what question is being answered? Is it to demonstrate this approach to citizen science data collection? Is it about orangutans, for which there an example of occupancy modelling? Is it about occupancy analysis more broadly? Is it the Living Plantation Index? There seems to be a mixture of all these and more. In many ways this reads like a project report for a funder rather than a planned scientific paper.

Response 1. We very much appreciate the constructive guidance from the editor and the reviewer and apologize for presenting a rather poorly structured manuscript that tried to present too many different aspects of our citizen science pilot study. Based on your feedback, we have significantly restructured the original manuscript and present a shorter and clearer revision. We have simultaneously submitted an additional manuscript to PLOS ONE that describes the occupancy analysis in much more detail, showing that the dataset is indeed robust. Incorporating that statistical analysis into the current manuscript would have made it too long, and probably also end up trying to target two distinct audiences, those interested in statistical modeling and those interested in practical solutions to wildlife monitoring in agricultural context.

We hope that the current manuscript is much more clearly structured around the four research questions at the end of the Introduction, with the sequence of these questions now being repeated in the Methods, Results and Discussion. We hope that with these changes, and a companion paper on the statistical models, we offer readers of PLOS ONE a much clearer and comprehensive overview of the potential value of citizen science-based wildlife monitoring in agricultural landscapes.

Comment 2. The occupancy modelling in particular seems out of place, yet also central to some messages. It is difficult to evaluate the Bayesian occupancy modeling since the details are absent and appear to be for a separate manuscript. Lines 127-128 – It is stated, “Detailed description of statistical methods and modelling assumptions for developing occupancy statistics from the PENDAKI data will be published elsewhere.” Also see lines 189-190. Why isn’t this part of the current manuscript?

Response 2. We considered incorporating the occupancy modelling into the manuscript, but this is a much more technical text that focuses on the specifics of the statistical models. Our current manuscript targets an audience of conservation practitioners working in palm oil or other agricultural contexts or for conservation NGOs. The second manuscript has a more scientific, and statistically trained target audience. We think it is better to present these two studies separately. We attach the second manuscript to this submission so that the editor and reviewer can access the information. We have also removed most references to the statistical analyses from the current manuscript as these are now addressed in the second manuscript.

Comment 3. The reviewer who would review the manuscript also had concerns about how it was written and the lack of clarity on the message. My suggestion is to decide exactly what the main message and goal of this manuscript is, remove the parts that are not relevant, and add anything that is absolutely necessary for a reviewer to understand and evaluate what you did. This can all potentially be fixed, but the authors will need to substantially rewrite what they have done. This may also result in the need for further review.

Response 3. Thank you for your guidance on this. We have significantly restructured the manuscript, with the 4 research questions at the end of the Introduction now sequentially addressed in the Methods, Results, and Discussion. As already mentioned, we have deleted the statistical modelling part and submitted a separate manuscript that describes this in much more detail.

Comment 4. The PLOS Data policy requires authors to make all data underlying the findings described in their manuscript fully available without restriction, with rare exception (please refer to the Data Availability Statement in the manuscript PDF file). The data should be provided as part of the manuscript or its supporting information, or deposited to a public repository. For example, in addition to summary statistics, the data points behind means, medians and variance measures should be available. If there are restrictions on publicly sharing data—e.g. participant privacy or use of data from a third party—those must be specified.

Response 4. We have made the raw data available at Zenodo pending final acceptance of the current manuscript: 10.5281/zenodo.15845290.

Reviewer #1: This is an interesting and important study - however I found that the presentation style needed significant improvement. The English language is correct, but the style is inappropriate for a journal paper. There is too much use of the passive tense, too many long sentences, a lot of relatively unimportant text is included and there is an absence of clear and direct statements of methods and results. Too much contextual text and a lot of text which only communicates options

Response 5. Thank you Dr Sayer; we greatly appreciate your feedback. We agree with you and the editor that the manuscript required significant rewriting and restructuring, which we have done. We have also removed information not directly relevant to the 4 research questions, and developed a separate manuscript on the occupancy modelling.

Attachment Submitted filename: Response to reviewers.docx

# Peer review: Decision Letter 1

**DOI:** 10.1371/journal.pone.0318046.r003

**Authors:** Bilal Habib

22 Feb 2026

Dear Dr. Meijaard,

Thank you for submitting your manuscript to PLOS ONE. After careful consideration, we feel that it has merit but does not fully meet PLOS ONE’s publication criteria as it currently stands. Therefore, we invite you to submit a revised version of the manuscript that addresses the points raised during the review process.

Please submit your revised manuscript by Apr 08 2026 11:59PM. If you will need more time than this to complete your revisions, please reply to this message or contact the journal office at plosone@plos.org. When you're ready to submit your revision, log on to https://www.editorialmanager.com/pone/ and select the 'Submissions Needing Revision' folder to locate your manuscript file.

- A letter that responds to each point raised by the academic editor and reviewer(s). You should upload this letter as a separate file labeled 'Response to Reviewers'.
- A marked-up copy of your manuscript that highlights changes made to the original version. You should upload this as a separate file labeled 'Revised Manuscript with Track Changes'.
- An unmarked version of your revised paper without tracked changes. You should upload this as a separate file labeled 'Manuscript'.

If you would like to make changes to your financial disclosure, please include your updated statement in your cover letter. Guidelines for resubmitting your figure files are available below the reviewer comments at the end of this letter.

If applicable, we recommend that you deposit your laboratory protocols in protocols.io to enhance the reproducibility of your results. Protocols.io assigns your protocol its own identifier (DOI) so that it can be cited independently in the future. For instructions see: https://journals.plos.org/plosone/s/submission-guidelines#loc-laboratory-protocols. Additionally, PLOS ONE offers an option for publishing peer-reviewed Lab Protocol articles, which describe protocols hosted on protocols.io. Read more information on sharing protocols at https://plos.org/protocols?utm_medium=editorial-email&utm_source=authorletters&utm_campaign=protocols.

We look forward to receiving your revised manuscript.

Kind regards,

Bilal Habib

Academic Editor

PLOS One

Journal Requirements:

If the reviewer comments include a recommendation to cite specific previously published works, please review and evaluate these publications to determine whether they are relevant and should be cited. There is no requirement to cite these works unless the editor has indicated otherwise.

Additional Editor Comments (if provided):

Please address the reviewer's comments. Since there was a delay in getting more reviews, I have taken time to review the manuscript. The detailed revision must include a clear response and changes to the manuscript regarding the following points.

This manuscript reports a five-year, large-scale rollout of a corporate citizen-science program to monitor biodiversity across multiple oil palm estates in Indonesia, and tests whether it can produce statistically meaningful wildlife data. It’s an ambitious, practically relevant effort, backed by a striking dataset: over 148,000 wildlife observations from nearly 4,000 contributors. The scope in time, space, and participation is a real strength. Methodologically, using Bayesian occupancy models for opportunistic citizen-science data makes sense, and the comparison between naïve species richness and occupancy-based estimates is genuinely informative. Importantly, the paper shows how biodiversity monitoring can be woven into plantation management at relatively low cost—a valuable applied contribution.

That said, the paper needs substantial revision before it’s ready for PLOS ONE. The biggest gap is methodological transparency. The manuscript notes that the technical details of the Bayesian occupancy models will be published elsewhere. That’s not sufficient for a standalone article. Readers need the full model structure, priors, treatment of detection probability, how uneven sampling effort was handled, spatial autocorrelation considerations, convergence diagnostics, and software specifics. Without these, the work isn’t reproducible and the robustness of the findings can’t be fairly assessed.

A related issue is sampling bias. Because observations are opportunistic and effort isn’t standardized, spatial and temporal biases are likely. Workers will tend to record species near roads, infrastructure, and frequently visited blocks; shifting reporting intensity over time could mimic trends. While occupancy models address imperfect detection, it’s not clear that observer heterogeneity, habitat-specific detectability, or variation in effort were adequately modeled. The paper would benefit from a sharper discussion here, and ideally a quantitative assessment of how these biases were diagnosed and mitigated.

The Living Plantation Index (LPI) is a creative idea, but its construction feels somewhat ad hoc. The weighting scheme that blends IUCN status, national protection, CITES listing, and range category needs either stronger theoretical grounding or evidence of robustness. A sensitivity analysis showing how alternative weights change the results would go a long way toward making the index scientifically defensible rather than merely communicative.

Some interpretations—especially around orangutan landscape use and corridor functionality—lean beyond what occupancy alone can support. Occupancy is not abundance, and non-detection is not proof of absence. These claims should be tempered to match the inferential limits of the modeling framework.

The interview-based social assessment is a useful complement but should be read cautiously given small samples, participant pre-selection, and possible social desirability bias. Likewise, sections highlighting ESG reporting gains and corporate recognition would benefit from a more neutral, scientific tone to avoid sounding promotional.

In sum, this is an important, large-scale attempt to integrate citizen science into corporate biodiversity management, with clear applied value and a promising analytical approach. To be publication-ready, it needs major revisions that: make the modeling fully transparent and reproducible; address sampling bias more rigorously; justify and stress-test the index; clarify potential conflicts of interest; and moderate conclusions to fit the evidence. With these improvements, the study could make a meaningful contribution to conservation monitoring and citizen science in production landscapes.

[Note: HTML markup is below. Please do not edit.]

Reviewers' comments:

Reviewer's Responses to Questions

**Comments to the Author**

Reviewer #2: (No Response)

**********

2. Is the manuscript technically sound, and do the data support the conclusions?

Reviewer #2: Yes

**********

3. Has the statistical analysis been performed appropriately and rigorously?

Reviewer #2: N/A

**********

4. Have the authors made all data underlying the findings in their manuscript fully available?

The PLOS Data policy

Reviewer #2: Yes

**********

5. Is the manuscript presented in an intelligible fashion and written in standard English?

Reviewer #2: Yes

**********

Reviewer #2: The work presented by the authors is truly inspiring and demonstrates an impressive integration of citizen participation in ecological monitoring. Your model of engagement and implementation provides valuable lessons for large-scale, community-driven research. The clarity of their methodological design makes this study a notable contribution to the growing field of citizen science.

That said, I would be very interested to see a more detailed discussion regarding how the authors addressed spatial and temporal biases inherent in citizen-science data collection. While occupancy models can indeed accommodate imperfect detection, they do not automatically correct for the non-random spatial distribution of effort or the temporal clustering of sampling events typical of volunteer-based monitoring. In such cases, detection probability may still be spatially autocorrelated with accessibility and human density, leading to systematic sampling. It would be good to write how you address those biases throe experiment design and data management.

Therefore, I would encourage the authors to elaborate on how their occupancy framework accounts for such structured bias.

In addition, I suggest situating the study within the broader landscape of citizen-science frameworks by discussing whether and how this work aligns with or diverges with other established models of citizen science.

Overall, this is a strong and well-executed manuscript. Addressing the two points above would further strengthen the paper and firmly position it within the wider discourse on citizen-science operational models.

**********

what does this mean?). If published, this will include your full peer review and any attached files.

If you choose “no”, your identity will remain anonymous but your review may still be made public.

**Do you want your identity to be public for this peer review?**  For information about this choice, including consent withdrawal, please see our Privacy Policy

Reviewer #2: No

**********

[NOTE: If reviewer comments were submitted as an attachment file, they will be attached to this email and accessible via the submission site. Please log into your account, locate the manuscript record, and check for the action link "View Attachments". If this link does not appear, there are no attachment files.]

To ensure your figures meet our technical requirements, please review our figure guidelines: https://journals.plos.org/plosone/s/figures

You may also use PLOS’s free figure tool, NAAS, to help you prepare publication quality figures: https://journals.plos.org/plosone/s/figures#loc-tools-for-figure-preparation.

NAAS will assess whether your figures meet our technical requirements by comparing each figure against our figure specifications.

# Author comment: Author response to Decision Letter 2

**DOI:** 10.1371/journal.pone.0318046.r004

10 Mar 2026

Editor comments

Comment 1. … the paper needs substantial revision before it’s ready for PLOS ONE. The biggest gap is methodological transparency. The manuscript notes that the technical details of the Bayesian occupancy models will be published elsewhere. That’s not sufficient for a standalone article. Readers need the full model structure, priors, treatment of detection probability, how uneven sampling effort was handled, spatial autocorrelation considerations, convergence diagnostics, and software specifics. Without these, the work isn’t reproducible and the robustness of the findings can’t be fairly assessed.

Response 1. Our target audience for the current paper is practitioners in the plantation industry. For that reason, we have kept the language in the manuscript non-technical and decided to publish the statistical model separately for readers who are interested in the statistical details. This paper has now been published in PLOS ONE and we refer to it in the current manuscript: https://journals.plos.org/plosone/article?id=10.1371/journal.pone.0328960. This paper is open access and therefore readers who are interested in the full model structure, use of priors, the use of detection probability to correct for sampling effort, and uneven spatial sampling and others, can refer to this online paper. Incorporating these model details into the current manuscript would make it unnecessarily lengthy and less suitable for a practitioner’s audience.

We hope that the editor agrees with us that there is value in providing practitioners in the palm oil industry with a broad overview of the use of citizen science-type approaches in wildlife monitoring in this highly scrutinized industry. We believe that the current manuscript meets PLOS ONE objectives of having a strong methodology in its detailed description of how the citizen science methods were implemented in the field, what the key elements were to its success, and what the practicalities are of using it in a palm oil context (uptake, cost, analytical output options, perceptions among work force).

Comment 2. A related issue is sampling bias. Because observations are opportunistic and effort isn’t standardized, spatial and temporal biases are likely. Workers will tend to record species near roads, infrastructure, and frequently visited blocks; shifting reporting intensity over time could mimic trends. While occupancy models address imperfect detection, it’s not clear that observer heterogeneity, habitat-specific detectability, or variation in effort were adequately modeled. The paper would benefit from a sharper discussion here, and ideally a quantitative assessment of how these biases were diagnosed and mitigated.

Response 2. The reviewer is right in saying that opportunistic data suffer from spatial and temporal biases; therefore, a statistical technique (occupancy modeling) was chosen that enables to consider these difficulties.

Occupancy models were originally designed to estimate true occupancy by correcting for differences in detection probability. But they can also be used as a tool to correct for unknown sampling effort. We explain in the separate paper mentioned above that this is a well-established model for analysis of citizen science data; see references in that paper). In short, the idea is that a higher sampling effort leads to a higher detection probability of a species within a block, provided the block is being occupied (production blocks in plantations were our unit of analysis). Blocks near roads and infrastructure are visited more frequently and less accessible blocks get fewer visits, which will lead to differences in reporting rate of a species between blocks. The model corrects for differences in the number of visits while estimating the occupancy probability of blocks. The same applies to changes in sampling effort over time. The model also distinguishes between the number of reported species – a longer list is often associated with higher sampling effort – , between observers to account for observer heterogeneity and between habitats to account for habitat-specific detection. In addition, obvious risks of spatial sampling bias were addressed in the companion paper. More particular, we tested whether blocks surveyed only once in 2020-2024 may lead to biased results. The results without these blocks, however, were not different than including them.

For these details on the statistical model, we refer the reader to the companion paper in PLOS ONE. The objective of the current paper is to convince our target audience of conservation practitioners and the agricultural sector that wildlife observations involving company work forces can usefully contribute to their adaptive management requirements. In our experience, this target audience is less interested in the statistical details than in practical questions about how to set up a program like this, cost considerations, and potential outputs from data analysis.

Comment 3. The Living Plantation Index (LPI) is a creative idea, but its construction feels somewhat ad hoc. The weighting scheme that blends IUCN status, national protection, CITES listing, and range category needs either stronger theoretical grounding or evidence of robustness. A sensitivity analysis showing how alternative weights change the results would go a long way toward making the index scientifically defensible rather than merely communicative.

Response 3. This is odd, because after the first round of reviews, we had removed the Living Plantation Index from the manuscript. In the revised version, there is no reference to this, and we wonder whether the editor has indeed seen the most up-to-date version of the manuscript. Could the editor please verify that the manuscript number PONE-D-24-59415_R1 was under review.

Comment 4. Some interpretations—especially around orangutan landscape use and corridor functionality—lean beyond what occupancy alone can support. Occupancy is not abundance, and non-detection is not proof of absence. These claims should be tempered to match the inferential limits of the modeling framework.

Response 4. Thank you for the comment.

We think that the make the distinction between occupancy and abundance quite clearly in the text. For example, in lines 337-340, we write “Orangutan occupancy in the KAL estate shows significant variation over the past 5 years, but we don’t know yet whether this truly reflects variation in orangutan abundance or behaviour, for example orangutans using oil palm areas more frequently when food was scarce.”

But we do not agree with the suggestion of the reviewer that we have only information about non-detections and not about absences of a species. Occupancy models enable to estimate the probability of real absences; these estimates are derived from non-detection records and the detection probability estimates.

Comment 5. The interview-based social assessment is a useful complement but should be read cautiously given small samples, participant pre-selection, and possible social desirability bias. Likewise, sections highlighting ESG reporting gains and corporate recognition would benefit from a more neutral, scientific tone to avoid sounding promotional.

Response 5. Thank you for the comment, and we have revised this text section to ensure a more neutral tone and more careful interpretation of the interview results. We also added a caveat in the Results section that draws attention to the small sample size and selection bias. Despite the small sample size and selection bias, we believe that the interviews provided important insights from partic

Comment 6. In sum, this is an important, large-scale attempt to integrate citizen science into corporate biodiversity management, with clear applied value and a promising analytical approach. To be publication-ready, it needs major revisions that: make the modeling fully transparent and reproducible; address sampling bias more rigorously; justify and stress-test the index; clarify potential conflicts of interest; and moderate conclusions to fit the evidence. With these improvements, the study could make a meaningful contribution to conservation monitoring and citizen science in production landscapes.

Response 6. Thank you for the supportive comments. We hope that the editor agrees that the companion paper with the statistical modelling details provides a suitable reference point for the readers to critically interpret the findings and claims in the current manuscript.

Comment 7. Reviewer #2: The work presented by the authors is truly inspiring and demonstrates an impressive integration of citizen participation in ecological monitoring. Your model of engagement and implementation provides valuable lessons for large-scale, community-driven research. The clarity of their methodological design makes this study a notable contribution to the growing field of citizen science.

That said, I would be very interested to see a more detailed discussion regarding how the authors addressed spatial and temporal biases inherent in citizen-science data collection. While occupancy models can indeed accommodate imperfect detection, they do not automatically correct for the non-random spatial distribution of effort or the temporal clustering of sampling events typical of volunteer-based monitoring. In such cases, detection probability may still be spatially autocorrelated with accessibility and human density, leading to systematic sampling. It would be good to write how you address those biases throe experiment design and data management.

Response 7. Thank you for your constructive feedback. As mentioned above, we decided to publish the statistical modeling details separately (https://journals.plos.org/plosone/article?id=10.1371/journal.pone.0328960).

In that study we used the same citizen science data discussed in the current manuscript.

See our Response 2 above how we dealt with the biases inherent in opportunistic data.

We understand that ideally these statistical details would have been covered in the current manuscript, but because we believe that our target audience for the current manuscript is more likely to read a less technical paper that describes the approach and its potential benefits in more general terms, leaving the statistical paper for readers who are more interested in methodological details.

Comment 8. In addition, I suggest situating the study within the broader landscape of citizen-science frameworks by discussing whether and how this work aligns with or diverges with other established models of citizen science.

Overall, this is a strong and well-executed manuscript. Addressing the two points above would further strengthen the paper and firmly position it within the wider discourse on citizen-science operational models.

Response 8.

Thank you for this helpful suggestion. We have added new text to the start of the Discussion that situates the PENDAKI program within commonly used citizen-science typologies. In particular, we note that citizen-science initiatives are often classified according to (i) the degree of participant involvement (e.g., contributory, collaborative, or co-created projects) and (ii) the structure of the monitoring design (structured, semi-structured, or opportunistic data collection). We clarify that PENDAKI most closely resembles semi-structured or opportunistic biodiversity monitoring systems in which observers record wildlife encountered during routine activities rather than through fixed survey protocols.

At the same time, we emphasize that PENDAKI differs from many citizen-science initiatives because participation occurs within a corporate workforce rather than among the general public. This creates a hybrid form of citizen science that combines elements of opportunistic biodiversity observation with aspects of workplace-based or community monitoring. We added text highlighting how this model leverages the continuous presence of estate workers across large landscapes, allowing biodiversity monitoring at spatial and temporal scales that would be difficult to achieve through conventional survey approaches. These additions help position the study within the broader citizen-science literature and clarify how the PENDAKI approach relates to existing frameworks.

Attachment Submitted filename: Response_to_reviewers_auresp_2.docx

# Editor report: Decision Letter 2

**DOI:** 10.1371/journal.pone.0318046.r005

**Authors:** Pratheep Annamalai

8 Jul 2026

Dear Dr. Meijaard,

Thank you for submitting your manuscript to PLOS ONE. The manuscript has improved substantially following revision with reviewers' comments. Now, after careful consideration, we feel that it has merit but does not fully meet PLOS ONE’s publication criteria as it currently stands. Therefore, we invite you to submit a revised version of the manuscript that addresses the points raised during the review process.

Please submit your revised manuscript by Aug 22 2026 11:59PM. If you will need more time than this to complete your revisions, please reply to this message or contact the journal office at plosone@plos.org. When you're ready to submit your revision, log on to https://www.editorialmanager.com/pone/ and select the 'Submissions Needing Revision' folder to locate your manuscript file.

- A letter that responds to each point raised by the academic editor and reviewer(s). You should upload this letter as a separate file labeled 'Response to Reviewers'.
- A marked-up copy of your manuscript that highlights changes made to the original version. You should upload this as a separate file labeled 'Revised Manuscript with Track Changes'.
- An unmarked version of your revised paper without tracked changes. You should upload this as a separate file labeled 'Manuscript'.

If you would like to make changes to your financial disclosure, please include your updated statement in your cover letter. Guidelines for resubmitting your figure files are available below the reviewer comments at the end of this letter.

If applicable, we recommend that you deposit your laboratory protocols in protocols.io to enhance the reproducibility of your results. Protocols.io assigns your protocol its own identifier (DOI) so that it can be cited independently in the future. For instructions see: https://journals.plos.org/plosone/s/submission-guidelines#loc-laboratory-protocols. Additionally, PLOS ONE offers an option for publishing peer-reviewed Lab Protocol articles, which describe protocols hosted on protocols.io. Read more information on sharing protocols at https://plos.org/protocols?utm_medium=editorial-email&utm_source=authorletters&utm_campaign=protocols.

As the corresponding author, your ORCID iD is verified in the submission system and will appear in the published article. PLOS supports the use of ORCID, and we encourage all coauthors to register for an ORCID iD and use it as well. Please encourage your coauthors to verify their ORCID iD within the submission system before final acceptance, as unverified ORCID iDs will not appear in the published article. *Only*  the individual author can complete the verification step; PLOS staff *cannot*  verify ORCID iDs on behalf of authors.

We look forward to receiving your revised manuscript.

Kind regards,

Pratheep K. Annamalai

Academic Editor

PLOS One

Journal Requirements:

1. If the reviewer comments include a recommendation to cite specific previously published works, please review and evaluate these publications to determine whether they are relevant and should be cited. There is no requirement to cite these works unless the editor has indicated otherwise.

2. Please review your reference list to ensure that it is complete and correct. If you have cited papers that have been retracted, please include the rationale for doing so in the manuscript text, or remove these references and replace them with relevant current references. Any changes to the reference list should be mentioned in the rebuttal letter that accompanies your revised manuscript. If you need to cite a retracted article, indicate the article’s retracted status in the References list and also include a citation and full reference for the retraction notice.

Additional Editor Comments:

Thank you for submitting your revised manuscript and for the considerable effort invested in addressing the previous reviewer and editorial comments. The manuscript has improved substantially. Before I can make a final decision, I would appreciate a small number of additional revisions aimed at improving clarity, transparency and overall alignment with the journal's style and scope.

1. First thing is around the description of the occupancy modelling approach. Acknowledging published methodology separately, the current manuscript's focus for a practitioner audience, many readers will only read the present paper separately. Therefore, it would be helpful to include a brief, non-technical summary of how the analyses accounted for issues such as detection probability, variation in observer effort, and differences among habitats and observers. A concise explanation would improve accessibility without substantially increasing the technical complexity of the manuscript.

2. Secondly around the tone, because current version of manuscript may be interpreted as promotional or advocacy-oriented lengthy awards discussion. Several statements describing the programme as a “success”, or linking it directly to improvements in the company’s environmental performance and reputation, would benefit from a more neutral scientific framing. The strength of the manuscript lies in the evidence presented, and the conclusions will be more convincing if they are expressed in a measured and objective manner. Similarly, authors can consider the discussion of awards and external recognition, to be shortened or reframed, so that it provides context rather than serving as evidence of programme effectiveness.

3. This is around response on online submission portal, declaring conflict of interests: Instead of sayign 'no competing interests, authors may mention "Several authors are employees of ANJ, the company where PENDAKI was implemented." Since the manuscript evaluates an ANJ-developed program, it may be a perceived conflict from editorial view (not technically).

4. The participation results are interesting and, yes, they represent one of the strengths of the paper. At the same time, manuscript reports that 'a relatively small proportion of contributors generated the majority of observations'. Authors consider to reflect on this point in the Discussion section, or to acknowledge that long-term biodiversity monitoring may depend heavily on a relatively small group of highly engaged participants. This would provide a balanced interpretation of programme uptake and sustainability.

5. This manuscript clearly shows that the programme can generate large quantities of useful biodiversity information, however, evidence (for programme directly improving biodiversity status or conservation outcomes) is relatively beyond the scope of the current study. Hence it can be toned down relevantly.

In gerenal, please undertake a final check of language, grammar, reference consistency and data availability information prior to submission.

[Note: HTML markup is below. Please do not edit.]

[NOTE: If reviewer comments were submitted as an attachment file, they will be attached to this email and accessible via the submission site. Please log into your account, locate the manuscript record, and check for the action link "View Attachments". If this link does not appear, there are no attachment files.]

To ensure your figures meet our technical requirements, please review our figure guidelines: https://journals.plos.org/plosone/s/figures

You may also use PLOS’s free figure tool, NAAS, to help you prepare publication quality figures: https://journals.plos.org/plosone/s/figures#loc-tools-for-figure-preparation.

NAAS will assess whether your figures meet our technical requirements by comparing each figure against our figure specifications.

# Author comment: Author response to Decision Letter 3

**DOI:** 10.1371/journal.pone.0318046.r006

3 Aug 2026

First thing is around the description of the occupancy modelling approach. Acknowledging published methodology separately, the current manuscript's focus for a practitioner audience, many readers will only read the present paper separately. Therefore, it would be helpful to include a brief, non-technical summary of how the analyses accounted for issues such as detection probability, variation in observer effort, and differences among habitats and observers. A concise explanation would improve accessibility without substantially increasing the technical complexity of the manuscript.

Response: We have inserted a paragraph that summarizes the occupancy methods and assumptions in simple words (Lines 193-205), while maintaining the reference to the van Strien et al. (2025) paper for technical details. We hope that this high-level description sufficiently clarifies the modelling approach for non-technical readers.

Secondly around the tone, because current version of manuscript may be interpreted as promotional or advocacy-oriented lengthy awards discussion. Several statements describing the programme as a “success”, or linking it directly to improvements in the company’s environmental performance and reputation, would benefit from a more neutral scientific framing. The strength of the manuscript lies in the evidence presented, and the conclusions will be more convincing if they are expressed in a measured and objective manner. Similarly, authors can consider the discussion of awards and external recognition, to be shortened or reframed, so that it provides context rather than serving as evidence of programme effectiveness.

Response: We thank the Editor for this helpful observation and agree that several sections of the manuscript could be interpreted as overly promotional. We have undertaken a thorough revision of the manuscript to adopt a more neutral scientific tone throughout. In particular, we have:

• removed evaluative terms such as successful, key to success, low-cost, important, critical, and empowered where these were not directly supported by the data;

• separated descriptive results from their interpretation, moving management implications and broader conclusions from the Results into the Discussion where appropriate;

• rewritten several Discussion sections so that interpretations are explicitly linked to interview findings or quantitative results rather than presented as general statements;

• shortened and reframed the discussion of awards and external recognition so that these provide context regarding external acknowledgement rather than evidence of programme effectiveness.

This is around response on online submission portal, declaring conflict of interests: Instead of sayign 'no competing interests, authors may mention "Several authors are employees of ANJ, the company where PENDAKI was implemented." Since the manuscript evaluates an ANJ-developed program, it may be a perceived conflict from editorial view (not technically).

Response: We agree and have changed the COI statement in the portal accordingly.

The participation results are interesting and, yes, they represent one of the strengths of the paper. At the same time, manuscript reports that 'a relatively small proportion of contributors generated the majority of observations'. Authors consider to reflect on this point in the Discussion section, or to acknowledge that long-term biodiversity monitoring may depend heavily on a relatively small group of highly engaged participants. This would provide a balanced interpretation of programme uptake and sustainability.

Response: We agree that this is an important point. We have expanded the Discussion to acknowledge that participation was highly skewed, with approximately 5% of observers contributing around 75% of all wildlife observations. We discuss this in the context of wider citizen science literature, noting that such participation patterns are common in volunteer-based monitoring programmes, and we consider the implications for long-term programme sustainability.

This manuscript clearly shows that the programme can generate large quantities of useful biodiversity information, however, evidence (for programme directly improving biodiversity status or conservation outcomes) is relatively beyond the scope of the current study. Hence it can be toned down relevantly.

Response: We agree and have revised the manuscript accordingly. We have removed or softened statements implying that PENDAKI directly improved biodiversity outcomes or conservation status. The revised manuscript now distinguishes more clearly between (i) evidence demonstrating the programme's capacity to generate biodiversity information and (ii) the potential management applications of these data, which are discussed as implications rather than demonstrated outcomes.

In general, please undertake a final check of language, grammar, reference consistency and data availability information prior to submission.

Response: In response to the Editor's comments, we also reviewed the structure of the manuscript to improve the separation between Results and Discussion. Descriptions of database management and validation procedures have been relocated to the Methods where appropriate, while interpretive statements and management implications have been transferred from the Results to the Discussion. This has resulted in a more objective presentation of the findings.

Attachment Submitted filename: Citizen Science in OIl Palm - response to comments.docx

# Editor report: Decision Letter 3

**DOI:** 10.1371/journal.pone.0318046.r007

**Authors:** Pratheep Annamalai

4 Aug 2026

Citizen science-based biodiversity monitoring in oil palm plantations

PONE-D-24-59415R3

Dear Dr. Meijaard,

We’re pleased to inform you that your revised manuscript has been reviewed again scientifically suitable for publication and will be formally accepted for publication once it meets all outstanding technical requirements.

Within one week, you’ll receive an e-mail detailing the required amendments. When these have been addressed, you’ll receive a formal acceptance letter and your manuscript will be scheduled for publication.

An invoice will be generated when your article is formally accepted. Please note, if your institution has a publishing partnership with PLOS and your article meets the relevant criteria, all or part of your publication costs will be covered. Please make sure your user information is up-to-date by logging into Editorial Manager at Editorial Manager® and clicking the ‘Update My Information' link at the top of the page. For questions related to billing, please contact billing support.

If your institution or institutions have a press office, please notify them about your upcoming paper to help maximize its impact. If they’ll be preparing press materials, please inform our press team as soon as possible -- no later than 48 hours after receiving the formal acceptance. Your manuscript will remain under strict press embargo until 2 pm Eastern Time on the date of publication. For more information, please contact onepress@plos.org.

Kind regards,

Pratheep K. Annamalai

Academic Editor

PLOS One

Additional Editor Comments (optional):

Thanks for addressing the comments.

Can you please check accuracy of references, particularly Ref 20?

Reviewers' comments:

# Editor report: Acceptance letter

**DOI:** 10.1371/journal.pone.0318046.r008

**Authors:** Pratheep Annamalai

PONE-D-24-59415R3

PLOS One

Dear Dr. Meijaard,

I'm pleased to inform you that your manuscript has been deemed suitable for publication in PLOS One. Congratulations! Your manuscript is now being handed over to our production team.

At this stage, our production department will prepare your paper for publication. This includes ensuring the following:

* All references, tables, and figures are properly cited

* All relevant supporting information is included in the manuscript submission,

* There are no issues that prevent the paper from being properly typeset

You will receive further instructions from the production team, including instructions on how to review your proof when it is ready. Please keep in mind that we are working through a large volume of accepted articles, so please give us a few days to review your paper and let you know the next and final steps.

Lastly, if your institution or institutions have a press office, please let them know about your upcoming paper now to help maximize its impact. If they'll be preparing press materials, please inform our press team within the next 48 hours. Your manuscript will remain under strict press embargo until 2 pm Eastern Time on the date of publication. For more information, please contact onepress@plos.org.

You will receive an invoice from PLOS for your publication fee after your manuscript has reached the completed accept phase. If you receive an email requesting payment before acceptance or for any other service, this may be a phishing scheme. Learn how to identify phishing emails and protect your accounts at https://explore.plos.org/phishing.

If we can help with anything else, please email us at customercare@plos.org.

Thank you for submitting your work to PLOS One and supporting open access.

Kind regards,

PLOS ONE Editorial Office Staff

on behalf of

Dr. Pratheep K. Annamalai

Academic Editor

PLOS One
